# Mass spectrometry‐based urinary proteomic analysis in feline chronic kidney disease

**DOI:** 10.14814/phy2.71072

**Published:** 2026-08-21

**Authors:** Jane H. C. Huang, Bianca N. Lourenço, Chad W. Schmiedt, Dennis R. Phillips

**Affiliations:** ^1^ Department of Small Animal Medicine and Surgery, College of Veterinary Medicine University of Georgia Athens Georgia USA; ^2^ Proteomics and Mass Spectrometry Facility, Department of Chemistry University of Georgia Athens Georgia USA

**Keywords:** domestic cat (*Felis catus*), random forest model, retinol‐binding protein 4 (RBP4), urinary biomarkers, urinary proteomic analysis

## Abstract

Chronic kidney disease (CKD) is highly prevalent in domestic cats (*Felis catus*), which might serve as a spontaneous disease model for human CKD. Urinary proteomics is a promising tool for CKD diagnosis and management; yet, minimal information is available in cats. This study aimed to explore the urinary proteome in cats with CKD and assess its diagnostic value. High‐performance liquid chromatography–tandem mass spectrometry (HPLC‐MS/MS) was used for proteomic analysis, and protein quantities were calibrated to a spike‐in internal standard. A cohort of 37 cats (14 with surgically induced CKD, 23 healthy controls) was used as a training dataset; a separate cohort of 40 cats (23 cats with naturally occurring CKD, 17 healthy controls) was used as the test dataset. A random forest model showed an area under the receiver‐operating‐characteristic (ROC) curve of 0.982 (95% CI, 0.952–1.000), outperforming serum symmetric dimethylarginine (SDMA) concentration, urinary protein‐to‐creatinine ratio, and other commonly studied urinary biomarkers (i.e., transferrin, albumin, and cystatin C). Retinol‐binding protein 4 (RBP4) achieved a comparable area under the ROC curve of 0.957 (95% CI, 0.885–1.000). These data provide proof of concept for a mass spectrometry‐based urinary proteomic analysis in feline CKD and identified urinary RBP4 as a CKD biomarker in cats.

## 1 INTRODUCTION

Chronic kidney disease (CKD) is highly prevalent in senior domestic cats (*Felis catus*), affecting 30%–80% of those greater than 10 years of age (Marino et al., 2014; Sparkes et al., 2016). The typical renal histological findings in cats with CKD (i.e., tubulointerstitial inflammation and fibrosis) (Brown et al., 2016) mirror those observed in people with some forms of CKD characterized by tubulointerstitial lesions (Devuyst et al., 2019; Gifford et al., 2017; Joyce et al., 2017; Weaver et al., 2015; Wijkström et al., 2017) and people with advancing CKD stages regardless of etiology (AlQudah et al., 2020; Sethi et al., 2017). Additionally, systemic arterial hypertension is reported in approximately 65% of cats with CKD—a prevalence comparable to that of people with CKD (Kalaitzidis & Elisaf, 2018; Muntner et al., 2010; Stiles, 1994). Because of these shared characteristics, cats have been proposed as a naturally occurring model for human CKD (Lawson et al., 2018; Lourenço et al., 2024); as companion animals, their physiological and environmental diversity also provides unique similarity to human beings that cannot be found in rodent models (Kol et al., 2015).

Urine is a source of renal‐specific biomarkers (Vlahou & Vanholder, 2026). While several urinary biomarkers of CKD have been investigated in cats, these candidates show significant heterogeneity within this population (Ferlizza et al., 2015; Kongtasai et al., 2022). A reasonable explanation is that urine production is a complex process affected by multiple factors, including glomerular permeability, tubular reabsorption and secretion, local (i.e., intrarenal) protein expression and secretion, and kidney cell damage or rupture. Given this complexity, it has been proposed that, relative to a single urinary marker, a urinary biomarker panel might more reliably reflect the status of kidney health (Kalantari et al., 2015). Proteomic analysis provides parallel screening of all proteins in a given library in a single run, making it suitable for constructing a multi‐marker panel, compared to assays that require pre‐determination of targeted proteins (e.g., immunoassay). It also provides a high‐resolution assessment of renal status that cannot be achieved using a single urinary biomarker, as well as insights into the physiological mechanisms underlying kidney disease.

To date, limited information is available for the urinary proteome of CKD in pet animals, including domestic cats and dogs. Published studies mostly used gel‐based protocols for protein quantification (Crisi et al., 2020; Giraldi et al., 2020), some with subsequent mass‐spectrometry‐based protein identification (Ferlizza et al., 2015; Ferlizza et al., 2020; Hormaeche et al., 2014; Lemberger et al., 2011; Nabity et al., 2011; Palviainen et al., 2012); this method, however, is limited by the fact that gel spots/bands and the proteins identified by these rarely showed a one‐to‐one relationship, challenging the legitimacy of using gel intensity for protein quantification. Quantification through tandem mass spectrometry has been the mainstay of urinary proteomic analysis in humans (Andersen et al., 2010; Fan et al., 2021; Lindhardt et al., 2017; Makhammajanov et al., 2024; Pontillo et al., 2017; Rossing et al., 2008). One previous study in cats used enhanced laser desorption‐ionization (SELDI) time‐of‐flight mass spectrometry for relative quantification of urinary proteins in cats (Jepson et al., 2013). While the protocol chosen could not identify protein sequences, eight molecules of unknown identity were found to predict the risk of azotemia, showing the potential of urinary proteomic analysis for early diagnosis and management of CKD in cats.

This study aimed to explore changes in the urinary proteome in cats with CKD and assess its diagnostic value. Urine from purpose‐bred, institution‐owned CKD cats was analyzed and used as a training set. The resulting model was then applied to urine collected from client‐owned cats (test set), and the abilities of the model or individual proteins to diagnose CKD in the test set were examined. Additionally, results from mass spectrometry‐based relative protein quantification were cross‐validated with an in‐house sandwich ELISA (Huang et al., 2026).

## 2 MATERIALS AND METHODS

### 2.1 Study design

**FIGURE 1:**
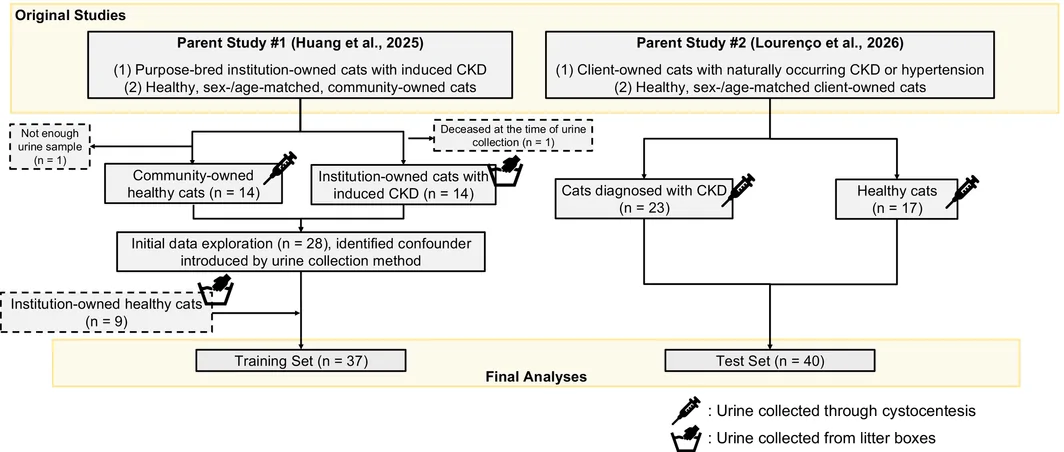
Overview of the study design. Cats were enrolled from two independent parent studies, consisting of a training set and a test set for final analyses. Parent Study #1 (Huang et al., 2025) included purpose‐bred institution‐owned cats with surgically induced chronic kidney disease (CKD) and healthy, sex‐ and age‐matched community‐owned cats. One CKD and one healthy cat were not enrolled due to being deceased before sample collection and a lack of a urine sample, respectively, resulting in 14 cats (8 spayed females, 6 castrated males) per group. During initial data exploration, a confounding effect from the urine collection method was identified. To resolve this confounder, additional group of institution‐owned healthy cats (*n* = 9; 5 spayed females and 4 castrated males) whose voided urine was collected from the litter box was enrolled, yielding a final training set of 37 cats (institution‐owned cats with induced CKD, *n* = 14; institution‐owned healthy cats, *n* = 9; community‐owned healthy cats, *n* = 14). From Parent Study #2 (Lourenço et al., 2026), client‐owned cats with naturally occurring CKD (*n* = 23; 14 spayed females and 9 castrated males) and healthy, sex‐ and age‐matched client‐owned cats (*n* = 17; 10 spayed females and 7 castrated males) were enrolled as the test set (total *n* = 40). All urine samples in the test set were collected via cystocentesis. CKD, chronic kidney disease.

This retrospective study used urine banked during two previous prospective studies (Huang et al., 2025; Lourenço et al., 2026) and from nine additional healthy cats. All cats from the parent studies with urine samples available at the start of this study (October 28, 2025) were enrolled. An overview of the study design is provided in Figure 1.

The training cohort included purpose‐bred cats with surgically induced CKD, sex‐ and life stage‐matched community‐owned healthy cats, and nine additional purpose‐bred healthy cats. Urine from the purpose‐bred cats with induced CKD and the matched control group was obtained and banked during a study designed to characterize the circulating renin‐angiotensinogen‐aldosterone system in cats with CKD (Huang et al., 2025). In those cats, CKD was induced by a two‐step 11/12th functional nephrectomy conducted from January to July 2019; the associated pathological and clinicopathological outcomes have been detailed in a prior publication describing the model (Schmiedt et al., 2023). By design of the parent study, urine from cats with induced CKD was collected from litter boxes lined with non‐absorbent litter between April 10 and 17, 2023, and urine from community‐owned healthy cats was collected via cystocentesis between September 9 and December 14, 2022. Additionally, urine from nine institution‐owned healthy cats was collected from litter boxes lined with non‐absorbent litter on February 26, 2026, to address differences in sample collection methods in the parent study, as will be described in the Section 2.2.

Urine collected for and banked during a previous study of client‐owned cats with naturally occurring CKD and age‐ and sex‐matched healthy controls was used as the test set (Lourenço et al., 2026). The parent study compared the circulating renin‐angiotensin‐aldosterone system in cats with non‐hypertensive CKD, treatment‐naïve hypertensive CKD, and normotensive healthy cats. For the current study, urine samples from CKD cats with or without systemic arterial hypertension were combined into a single CKD group.

In all instances, urine collected for prior studies was aliquoted and frozen at −80°C for future analysis.

### 2.2 Animals and sample collection

All cats enrolled as the control group of this study, including the control group of the two parent studies (Huang et al., 2025; Lourenço et al., 2026) and the nine additional healthy cats, were deemed healthy based on medical history, physical examination, and clinicopathological assessment (hematologic, serum biochemical, and urinalysis evaluations, including urinary protein‐to‐creatinine ratio [UPC]).

#### 2.2.1 Animals and sample collection, training set

For all purpose‐bred cats (cats with surgically induced CKD and the 9 additional healthy cats), urine was collected from a litter box filled with non‐absorbent plastic beads. A detailed description of cats with surgically induced CKD is available in the parent study (Huang et al., 2025); at the time of urine sample collection, all cats with surgically induced CKD were clinically stable. The nine additional healthy cats were purpose‐bred cats maintained by our research team for a separate project. These cats were obtained from a commercial source (Marshall Bioresources; North Rose, NY, USA) on November 5th, 2025, and are kept in the same environment as the institution‐owned CKD cats as previously described (Schmiedt et al., 2023), except they are fed a maintenance adult diet (Cat Chow, Purina; St. Louis, MO, USA) instead of therapeutic renal diet.

For client‐owned cats in the control group of the parent study (Huang et al., 2025), urine was collected through cystocentesis. These cats were privately owned by staff or students at the University of Georgia Veterinary Teaching Hospital (Athens, GA, USA) and were enrolled between September 9 and December 14, 2022. Enrollment was based on sex and life‐stage matching to the surgically induced CKD cohort. Written consent was obtained from all owners prior to participation.

All experimental procedures were approved by the University of Georgia Institutional Animal Care and Use Committee (protocol No. A2018 10‐001 and A2025 07‐019) and the Clinical Research Committee of the University of Georgia College of Veterinary Medicine (approval No. CR‐665).

#### 2.2.2 Animals and sample collection, test set

Descriptions of the case criteria, enrollment process, and resulting cat population of the test cohort are available in the parent study (Lourenço et al., 2026). Briefly, client‐owned cats presenting to the University of Georgia Animal Teaching Hospital (Athens, GA, USA) were enrolled between March 30, 2021 and September 18, 2023. Cats in the CKD group were diagnosed with International Renal Interest Society stage 2 or higher (i.e., serum creatinine concentration ≥1.6 mg/dL with contemporaneous urine specific gravity <1.035 and absence of apparent extra‐renal cause). Cats in the control group were enrolled based on age, sex, and neuter status to match those of the CKD group.

### 2.3 Sample analysis

#### 2.3.1 Urine sample pretreatments

Urine total protein concentration was measured using a turbidimetric method by the University of Georgia Veterinary Teaching Hospital Clinical Pathology Laboratory (Athens, GA, USA). For each sample, a volume corresponding to 0.05 mg of total protein was used for mass‐spectrometry‐based urinary proteomic analysis. Five hundred nanograms of alcohol dehydrogenase (ADH1) from *Saccharomyces cerevisiae* (Sigma‐Aldrich [RRID:SCR_008988], Catalog# A8656; St. Louis and Burlington, MA, USA) was spiked into each sample as an internal standard. The mixture was then heated at 95°C for 10 min in 15 mM dithiothreitol (Thermo Fisher Scientific [RRID:SCR_008452] Catalog# R0861; Waltham, MA). Samples were then loaded onto 10 kDa filters (Sartorius [RRID:SCR_003935] item# VN01H02; Göttingen, Germany), spun down to almost dry at 14000 g, then washed with 450 μL 20 mM triethylammonium bicarbonate buffer (Sigma‐Aldrich [RRID:SCR_008988], Catalog# T7408‐100ML; St. Louis and Burlington, MA, USA) three times. After the last wash, the filter columns were transferred to new collecting tubes, and 0.001 mg of trypsin (Promega [RRID:SCR_006724], Catalog# V5113; Madison, WI, USA) in triethylammonium bicarbonate buffer was added (protein‐trypsin w/w ratio = 1:50). Samples were incubated at 37°C overnight. The next day, digested samples were spun down into new collection tubes, followed by two washes with 100 μL 0.1% formic acid (Thermo Fisher Scientific [RRID: SCR_008452] Catalog# 270480250; Waltham, MA, USA). The flow‐through was collected.

#### 2.3.2 Mass spectrometry urine proteomic analysis

Samples were dried using a SpeedVac, then resuspended in 25 μL 25% acetonitrile in 0.1% formic acid (Honeywell Catalog# 441‐4; Charlotte, NC, USA). The resuspended samples were separated by a high‐performance liquid chromatography system (Bruker Elute UHPLC; Billerica, MA, USA) with a C18 wide pore (2.6 μm) column (ChromaNik Sunshell C18‐WP, Catalog# CW6971; Osaka, Japan). The column was held at 40°C, and mobile phase consisted of 0.1% formic acid in water (Sigma‐Aldrich [RRID:SCR_008988], Catalog#5.33002; St. Louis and Burlington, MA, USA) and acetonitrile in 0.1% formic acid (Honeywell Catalog# 441‐4; Charlotte, NC, USA), with the latter went from 5% to 10% in the first 5 min; 10% to 22% in the fifth to 38th minutes; 22 to 40% from 38th to 57th minutes; 40% to 100% from 57th to 62nd minutes; held at 100% for 3 min, then down from 100 to 5% in the 65th to 70th minutes (the last 5 min of each run). The flow rate was 200 μL per minute, and the column was equilibrated for 5 min between each run.

Spectra were acquired using a quadrupole time‐of‐flight (Q‐TOF) instrument (Bruker Impact II; Billerica, MA, USA) in the 3rd–58th minutes of each run. The instrument capillary was set at 3500 V, the nebulizer at 2 Bar, dry gas (nitrogen) at 6 L/min, and a dry temperature at 200°C. For precursor ion selection, the instrument scanned from 80 to 2800 mass‐to‐charge ratio at 10 Hz, with a threshold of 650 counts and a maximum of 12 precursors allowed at each scan. Active exclusion was enabled after 1 spectrum and released after 0.5 min, but allowed re‐entry when the new signal was >10 times greater than the previous signal. Two and 3 charged states were preferred, while singly charged ions were allowed when fewer than 12 ions with 2 or 3 charges passed the threshold. Mass‐to‐charge ratios in the calibration fluid (Bruker TuneMix; Billerica, MA, USA) were actively excluded. The product ion detection was set at 6 Hz. Data were saved in line (centroid) spectra only.

#### 2.3.3 Angiotensinogen ELISA

Concentrations of angiotensinogen in samples from two parent studies (Huang et al., 2025; Lourenço et al., 2026) were measured using an in‐house ELISA specifically designed for feline samples (Huang et al., 2026). Measurements and data analyses were conducted in accordance with the procedure described in the method validation study, including running all samples in triplicate and using the average of the three measurements. To compare with the results from mass spectrometry proteomic analysis, concentrations obtained from ELISA were divided by the total protein amount of the sample and expressed in nanograms of angiotensinogen per milligram of total protein.

### 2.4 Statistical analysis

An open‐source software, MaxQuant (RRID:SCR_014485; version 2.6.4.0), was used for protein identification and quantification (Tyanova, Temu, & Cox, 2016). Output from MaxQuant was read into R Project for Statistical Computing (RRID:SCR_001905; version 4.5.1), in which the following data processing was conducted. For statistical purposes, urine specific gravity >1.060 was computed as 1.061.

As the total protein amount in each sample was standardized (as described in the sample analysis—Section 2.3.1), quantification results represent the relative abundances of proteins (i.e., the proportion that protein accounted for within the total urinary protein) and will be referred to as “abundance” in the following text.

#### 2.4.1 Proteomic data processing

Proteins belonging to the domestic cat (*Felis Catus*) from UniProt (RRID:SCR_002380) were used as the reference database (accessed on June 1st, 2025). Methionine oxidation and N‐terminal acetylation were set as variable modifications. Search for potential contaminants was enabled, and decoy mode was set to “Revert” (i.e., reverse sequences). Digestion method was set at Trypsin/P (trypsin cleavage allowing following proline); minimum peptide length was 7, and up to 2 missing cleavages were allowed. All other parameters were left as default, including a false‐discovery rate of 0.01 at peptide‐spectrum match, peptide discovery, and protein discovery.

Output from MaxQuant was read into R Project for Statistical Computing (RRID:SCR_001905; version 4.5.1). Protein abundance was obtained from the “Top 3” output of MaxQuant (i.e., from the summed intensity of the top 3 peptides for each protein) (Silva et al., 2006); this method is considered well‐suited to our samples given the wide dynamic range (Ahrné et al., 2013). Proteins that were identified as potential contaminants, reverse hits, or only identified by site were excluded. Protein abundances were log_2_‐transformed to approximate a normal distribution, calibrated using the internal standard (ADH1), and subsequently *z*‐score normalized.

Quality control and data imputation were developed using the training data alone, before being applied to both the training and test data. For quality control, only proteins identified in >50% of samples in at least one of the disease groups (i.e., in >50% of samples in the control or CKD group) in the training data entered further analysis. For the included proteins, missing values were imputed based on the assumption of missing‐not‐at‐random (i.e., proteins were not observed due to a low concentration). Values were drawn from a left‐shifted normal distribution built using training data, with the default parameters used by the open‐source software Perseus (Tyanova, Temu, Sinitcyn, et al., 2016). For each protein, the mean and standard deviation (SD) of observed values were calculated, and a new normal distribution was generated using a new mean and SD computed from the original ones:

$${\text{Mean}}_{\mathrm{new}}={\text{Mean}}_{\text{observed}}-\left(1.8\times {\mathrm{SD}}_{\text{observed}}\right)$$

$${\mathrm{SD}}_{\mathrm{new}}=0.3\times {\mathrm{SD}}_{\text{observed}}$$

Missing values for each protein were imputed based on the computed new distribution. The imputed data were used for all the following statistical analyses.

#### 2.4.2 Training data characterization

Principal component analysis (PCA) was conducted using the R base package stats. Correlations between angiotensinogen measured by ELISA and by mass spectrometry were analyzed using Spearman correlation analysis. Logistic or linear models were built for each protein, with protein abundance set as the independent variable, and disease group or clinical parameters (systolic blood pressure [SBP], serum creatinine, or UPC) as the outcome. The *p*‐values were then adjusted for multiple comparisons using the Benjamini‐Hochberg false discovery rate (FDR) method. For the logistic regression analysis comparing protein abundances between groups, a >2‐fold difference in mean protein abundance, together with an adjusted *p*‐value < 0.05, was considered meaningful. For the linear regression analysis, the association was considered meaningful if the adjusted *p*‐value is less than 0.05, and a two‐fold change in urinary protein abundance corresponds to >10 mmHg difference in SBP, >0.3 mg/dL difference in serum creatinine concentration, or >0.1 difference in UPC.

#### 2.4.3 Model training and evaluation

Using the training data, a Random Forest classifier was built to predict the study group (control versus CKD) with R package caret (version 7.0.1) (Kuhn, 2008). Optimal hyperparameters were identified with 5‐fold cross‐validation, with all other parameters left as default.

Model performance was evaluated on the out‐of‐bag samples from the training set and on the test set. Receiver‐operating curve (ROC) and an optimal cutoff for CKD probability, defined based on the Youden index (sensitivity + specificity), were computed using R package pROC (version 1.19.0.1) (Robin et al., 2011), with 95% confidence interval (CI) computed using DeLong's method (the package's default setting). Proteins' contribution to the models was examined based on their overall importance, as calculated by the function varImp in the R package caret. For comparing the model's performance to that of single biomarkers, ROCs were also computed for all individual proteins that were more abundant in training set's CKD group.

In addition to the full protein set, performance of a subset of proteins chosen by elastic net was evaluated. The elastic net was designed using the R package glmnet (version 5.0) (Friedman et al., 2010) and caret (version 7.0.1) (Kuhn, 2008). Two parameters: alpha (for balance between ridge and lasso regression) and lambda (for weighting the penalty of including more variables) were tuned. Alpha was tested at 11 evenly spaced values from 0 to 1 (in increments of 0.1), and lambda was tested at 100 evenly spaced values from 0.001 to 1, yielding 1100 candidate hyperparameter combinations. Model performance for each combination was evaluated using 5‐fold cross‐validation repeated 5 times, and the combination maximizing cross‐validated accuracy was selected as the final model. The process of training and testing the random forest model was then repeated, using only the subset of proteins selected by elastic net.

## 3 RESULTS

### 3.1 Training data characteristic

One community‐owned healthy cat from the parent study (Huang et al., 2025) did not have sufficient volume of urine at the start of this study, and one cat with induced CKD was deceased at the point of urine collection as described in the parent study. This left 14 cats in the induced CKD group and 14 in the community‐owned control group. An initial exploration of samples from the parent study revealed that hemoglobin subunits (UniProt ID, P07412 and P07405) were substantially higher in urine from the healthy group, while major allergen I polypeptide chain 1 (UniProt ID, P30438), a protein that predominantly exists in cats' fur and saliva, was substantially higher in urine from the CKD group (Figure S1). We considered this likely to stem from a difference in urine collection method (cystocentesis in the healthy group, versus free‐catch from litter boxes in the CKD group). Additional voided samples were therefore collected from litter boxes of nine other institution‐owned, purpose‐bred healthy cats, as described in the Section 2.

The final training data, therefore, included 14 institution‐owned cats with surgically induced CKD; 14 community‐owned healthy cats; and nine institution‐owned, purpose‐bred healthy cats (Table 1; Table S1).

**TABLE 1:** Characteristics of the training cohort.

	CKD, voided (*n* = 14)	Control, cystocentesis (*n* = 14)	Control, voided (*n* = 9)	*p*‐value
Age (year)	5.08 [5.05; 5.10]^a^	3.34 [2.43; 3.55]^b^	2,85 [2.83; 2.92]^b^	<0.001
Sex				
Female, Spayed	8 (57.1%)	8 (57.1%)	5 (55.6%)	>0.999
Male, Castrated	6 (42.9%)	6 (42.9%)	4 (44.4%)	
Serum Creatinine (mg/dL) RI, 0.7–1.8 mg/dL	2.1 [1.9; 2.4]^a^	1.5 [1.2; 1.6]^b^	1.4 [1.2; 1.4]^b^	<0.001
Serum SDMA RI, 0–14 𝜇g/dL	Not measured	12 [10; 13]	13 [13; 14]	0.017
Blood Urea Nitrogen (mg/dL) RI, 18–35 mg/dL	22 [21; 26]	23 [20; 26]	21 [18; 24]	0.570
Urine‐Specific Gravity	1.033 [1.022; 1.042]	1.051 [1.045; 1.053]	1.058 [1.054; 1.061]	<0.001
UPC	0.11 [0.07; 0.14]	0.11 [0.09; 0.16]	0.10 [0.08; 0.12]	0.233
Urinary Protein (mg/dL)	24 [15; 36]	34 [29; 46]	41 [28; 52]	0.043
Urinary Creatinine (mg/dL)	222 [134; 299]^a^	305 [261; 353]^a^	409 [378; 474]^b^	0.001

*Note*: Continuous or categorical variables were reported in median [interquartile range] or *n* (%), respectively. *p*‐values were computed with the Kruskal–Wallis test for continuous variables and Fisher's exact test for categorical variables. Pairwise comparisons were adjusted using the Benjamini and Hochberg method. ^a,b^: Groups sharing a superscript letter are not significantly different at a 5% significance level.

Abbreviations: RI, reference interval; SDMA, Symmetric dimethylarginine; UPC, urinary protein‐to‐creatinine ratio.

### 3.2 Urinary proteomic analysis in training data

Six‐hundred‐thirty‐seven proteins were identified across the training data set after excluding spike‐in standard and proteins marked as potential contaminants, reverse hits, or only identified by site in MaxQuant. Among them, 266 proteins were identified in at least 50% of samples in the control or CKD group and entered the final analysis.

**FIGURE 2:**
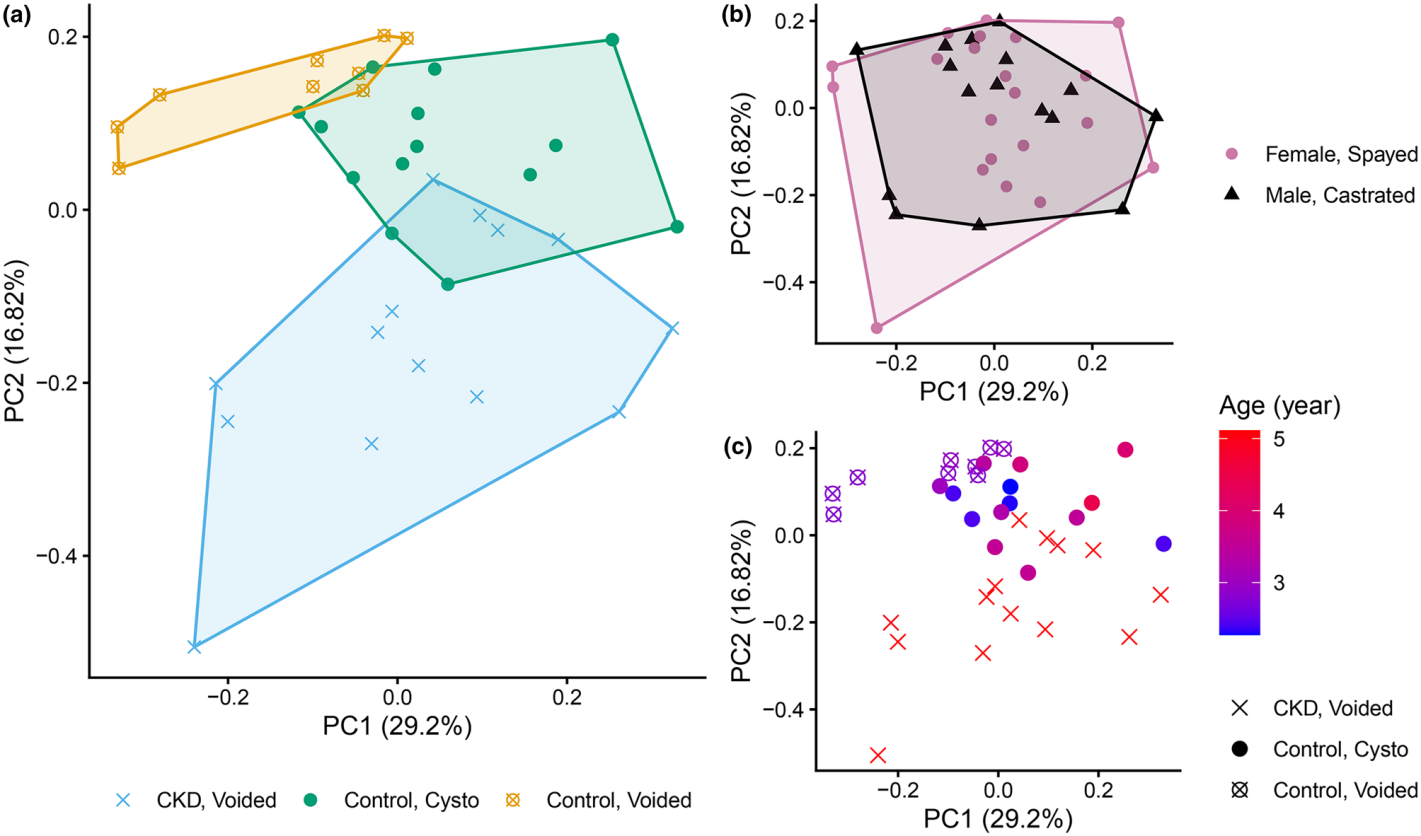
Principal component analysis (PCA) was performed on urinary proteomic data from the training set (*n* = 37; 21 spayed females, 16 castrated males). Each data point represents one individual cat. (a) Cats are color‐coded and grouped by collection method and disease status: CKD cats whose urine was collected via free‐catch from litter boxes (CKD, Voided; blue crosses; *n* = 14), community‐owned healthy control cats whose urine was collected via cystocentesis (Control, Cysto; green filled circles; *n* = 14), and healthy control cats whose urine was collected via free‐catch from litter boxes (Control, Voided; yellow crossed circles; *n* = 9). (b) The same PCA plot with cats color‐coded by sex: Spayed females (pink circles; *n* = 21) and castrated males (black triangles; *n* = 16), indicating no appreciable separation by sex. (c) The same PCA plot with point color indicating age (years; continuous color scale from blue [youngest] to red [oldest]) and point shape indicating collection method and disease status: CKD, voided (crosses); Control, cystocentesis (filled circle); Control, voided (crossed circle). No systematic clustering by age is apparent. CKD, chronic kidney disease; Cysto, cystocentesis; Voided, urine collected from litter boxes; PC(A), principal component (analysis).

Principal component analysis (PCA) revealed clustering based on disease status (CKD versus control) along the second principal component (PC2) (Figure 2a). In our study population that consists exclusively of sterile young adults (Quimby et al., 2021), the first two principal components were not driven by sex or age (Figure 2b,c).

**FIGURE 3:**
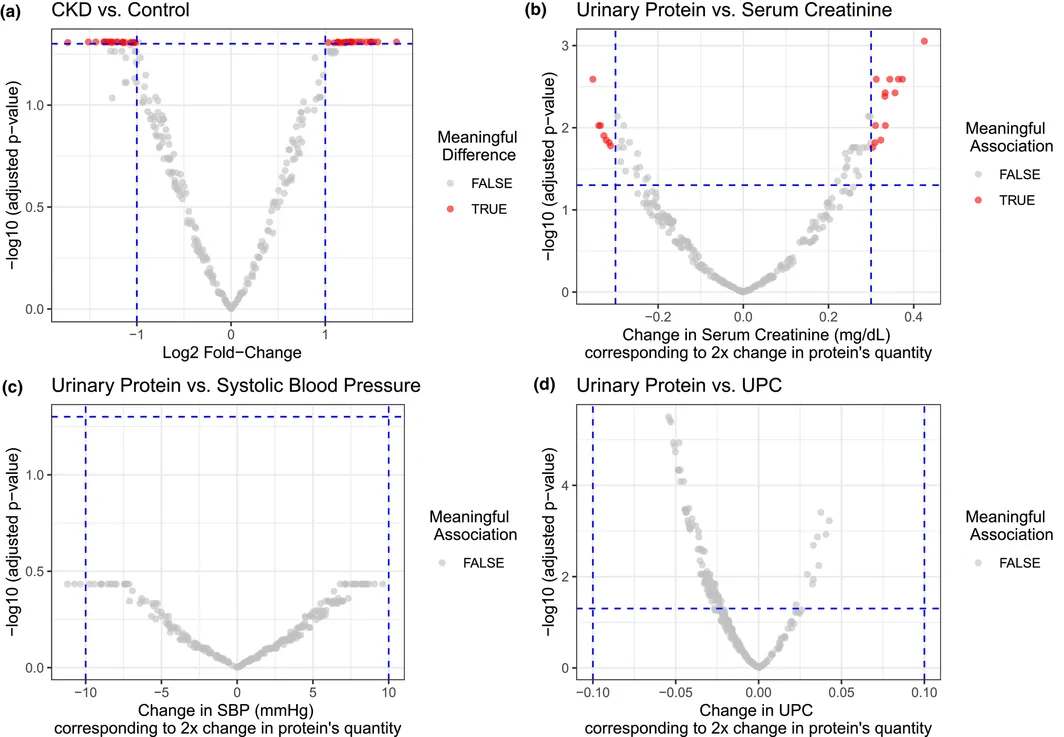
Volcano plots for 266 urinary proteins' differential abundance and association with clinical parameters in the training set (*n* = 37; CKD cats, *n* = 14; healthy cats, *n* = 23). Each point represents one urinary protein. The y‐axis shows the −log_10_ FDR‐adjusted *p*‐value; the horizontal dashed blue line indicates the threshold for statistical significance (FDR‐adjusted *p*‐value < 0.05). The x‐axis shows the effect size; vertical dashed blue lines indicate the respective effect size thresholds for each analysis. Proteins that meet both the significance and effect size thresholds and are designated as showing a meaningful difference (panel a) or association (panels b–d) and are highlighted in red. (a) Logistic regression analysis comparing urinary protein abundance between cats with CKD and healthy controls. The effect size threshold was set at log_2_ fold‐change = ±1 (i.e., a 2‐fold change between groups). Fourty‐eight proteins were found to be meaningfully different between groups; 26 were higher, and 22 were lower in the CKD group. (b) Linear regression analysis for the association between urinary protein abundance (independent variable) and serum creatinine concentration (dependent variable). The effect size threshold was set at >0.3 mg/dL difference in serum creatinine per two‐fold change in the protein's abundance. Twenty proteins were found to be meaningfully associated with serum creatinine (13 positively, 7 negatively). (c, d) Linear regression analysis for the association between urinary protein abundance (independent variable) and SBP or UPC (dependent variable). The effect size threshold was set at >10 mmHg or >0.1 difference in SBP or UPC per two‐fold change in the protein's abundance. No proteins were found to be meaningfully associated with SBP or UPC. FDR, false‐discovery rate; SBP, systolic blood pressure; UPC, urinary protein‐to‐creatinine ratio.

Logistic regression revealed 48 proteins that differed between CKD and control groups by more than 2‐fold and with FDR‐adjusted *p*‐value < 0.05. Among them, 26 were higher, and 22 were lower in the CKD group; notably, urinary cubilin was lower in cats with CKD, with a mean abundance of 0.350‐fold compared to that of healthy cats (Figure 3a, Table S2). Under linear regression analysis, 20 proteins associated with serum creatinine (13 positively, 7 negatively) with adjusted *p*‐value < 0.05 and magnitude of >0.3 mg/dL per two‐fold change of the protein (Figure 3b, Table S3). No protein was identified as meaningfully associated with SBP or UPC as defined in the Section 2.4 (Figure 3c,d).

### 3.3 Random forest model for disease group prediction

A random forest model was developed using the training data. Internal validation was performed on out‐of‐bag samples. At a decision threshold of 49.76%, determined by maximizing the Youden index, the model achieved a sensitivity of 92.9% and a specificity of 95.7%, with an area under the curve (AUC) of 0.969 (95% CI, 0.921–1.000) under ROC analysis, outperforming UPC and close to the performance of serum creatinine concentration in the training set (Figure S2).

**FIGURE 4:**
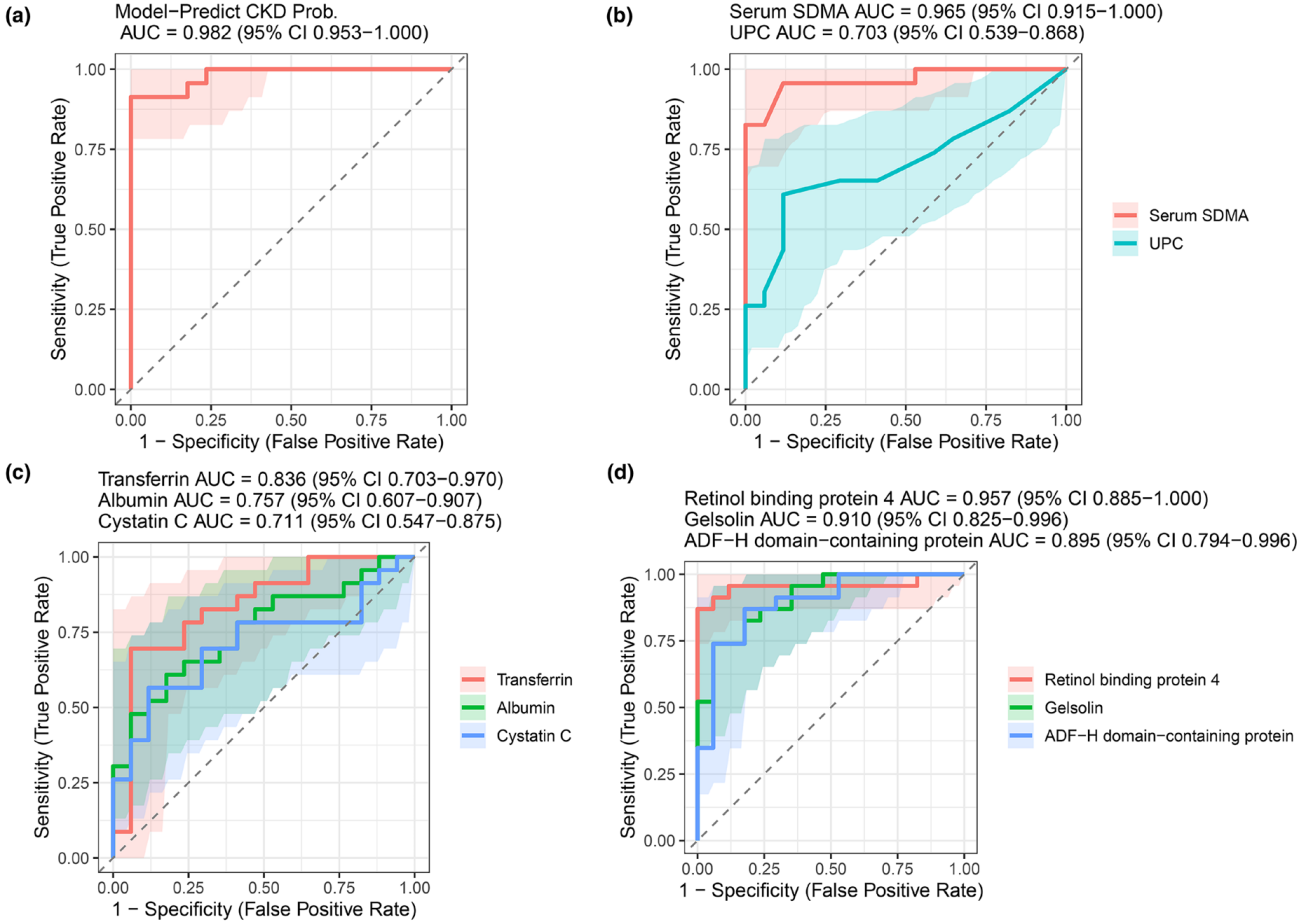
Receiver operating characteristic (ROC) curve analysis in the test set (*n* = 40; 23 cats with naturally occurring CKD, 17 healthy cats). Analysis was conducted using the R package pROC (version 1.19.0.1); shaded regions around each curve represent the 95% confidence interval computed using DeLong's method. (a) ROC curve for the random forest model‐predicted CKD probability (AUC = 0.985; 95% CI 0.959–1.000). (b) ROC curves for two conventional clinical renal biomarkers: Serum symmetric dimethylarginine (SDMA; AUC = 0.965; 95% CI 0.915–1.000; pink) and urinary protein‐to‐creatinine ratio (UPC; AUC = 0.703; 95% CI 0.539–0.868; sage). (c) ROC curves for urinary proteins previously investigated as renal biomarkers: Transferrin (AUC = 0.836; 95% CI 0.703–0.970; red), albumin (AUC = 0.749; 95% CI 0.596–0.902; green), and cystatin c (AUC = 0.711; 95% CI 0.547–0.875; blue). (d) ROC curves for three urinary proteins showing the highest AUCs among all proteins. Retinol binding protein 4 (AUC = 0.977; 95% CI 0.941–1.000; pink), gelsolin (AUC = 0.910; 95% CI 0.825–0.996; green), and ADF‐H domain‐containing protein (AUC = 0.895; 95% CI 0.794–0.996; blue). ADF‐H, actin‐depolymerizing factor homology; AUC, area under curve; CI, confidence interval; CKD, chronic kidney disease; SDMA, symmetric dimethylarginine; UPC, urinary protein‐to‐creatinine ratio.

For the test cohort, all animals from the parent study had sufficient urine samples and were included, resulting in 23 client‐owned cats with naturally occurring CKD and 17 healthy cats (Lourenço et al., 2026) (Table 2; Table S4). The model was applied to the test data set. In ROC analysis, the model achieved an AUC of 0.982 (95% CI, 0.953–1.000), with the optimal cutoff of 54.7% yielding 91.3% sensitivity and 100% specificity for CKD diagnosis (Figure 4a), outperforming UPC and serum SDMA (Figure 4b) and urinary biomarkers that have previously been proposed, including transferrin, albumin, and cystatin C (Figure 4c).

**TABLE 2:** Characteristics of the test cohort.

	CKD, cystocentesis (*n* = 23)	Control, cystocentesis (*n* = 17)	*p*‐value
Age (year)	13.5 [12.8; 16.2]	11.6 [9.79; 14.1]	0.020
Sex			
Female, Spayed	14 (60.9%)	10 (58.8%)	>0.999
Male, Castrated	9 (39.1%)	7 (41.2%)	
Serum Creatinine (mg/dL) RI, 0.7–1.8 mg/dL	2.1 [1.9; 2.8]	1.1 [1.0; 1.3]	Not applicable^a^
Serum SDMA RI, 0–14 𝜇g/dL	18 [15; 22]	11 [9; 11]	Not applicable^a^
BUN (mg/dL) RI, 18–35 mg/dL	39 [34; 50]	23 [22; 25]	<0.001
Urine‐Specific Gravity	1.019 [1.016; 1.024]	1.053 [1.048; 1.057]	<0.001
UPC	0.18 [0.12; 0.28]	0.13 [0.11; 0.16]	0.030
Urinary Protein (mg/dL)	21 [12; 32]	39 [29; 50]	0.001
Urinary Creatinine (mg/dL)	104 [72.3; 139]	290 [253; 319]	<0.001

*Note*: Continuous or categorical variables were reported in median [interquartile range] or *n* (%), respectively. *p*‐values were computed with the Kruskal–Wallis test for continuous variables and Fisher's exact test for categorical variables.

Abbreviations: RI, reference interval; SDMA, Symmetric dimethylarginine; UPC, urinary protein‐to‐creatinine ratio.

^a^ *p*‐values were not computed due to variables being part of the grouping criteria.

The overall importance of specific proteins for the model decision was examined (Table S5). Among the 266 proteins used for model training, 115 proteins had higher mean abundance in the CKD group in the training cohort (i.e., higher in urine from cats with surgically induced CKD, compared to the training cohort control group). When used for diagnosing naturally occurring CKD in the test dataset, these proteins yielded AUCs ranging from 0.435 to 0.957 (Table S6). The ROC curves for the 3 proteins with the highest AUCs are shown in Figure 4, panel D. Retinol binding protein 4 (UniProt ID: M5AXY1), a protein that has been previously studied as a potential urinary biomarker, showed the highest AUC of 0.957 (95% CI, 0.885–1.000), with an optimal cutoff yielding 100% specificity and 87.0% sensitivity.

### 3.4 ELISA cross‐validation

**FIGURE 5:**
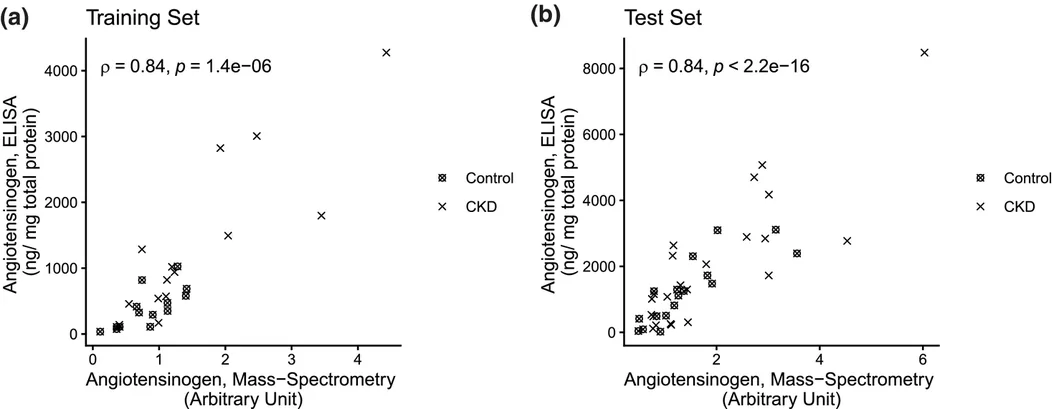
Urinary angiotensinogen abundance measured by mass spectrometry versus ELISA. Urinary angiotensinogen abundance was measured using a previously validated in‐house ELISA (Huang et al., 2026) for urine samples from parent studies (Huang et al., 2025; Lourenço et al., 2026), and the results (y‐axis; ng/mg total protein) were compared with those measured by mass spectrometry (x‐axis; arbitrary units, normalized to a spike‐in internal standard). Each data point represents one individual cat (CKD cats, cross; control cats, crossed circle). Correlation was assessed using Spearman's correlation analysis. (a) Training set: ELISA was conducted on samples from the parent study (*n* = 28; 14 institution‐owned cats with induced CKD, 14 community‐owned healthy controls). Spearman's *ρ* = 0.84, *p*‐value < 0.001. (b) Test set (*n* = 40; 23 client‐owned cats with CKD; 17 community‐owned healthy cats). Spearman's *ρ* = 0.84, *p*‐value < 0.001. CKD, chronic kidney disease.

To validate the mass spectrometry‐based protein quantification, the abundance of the protein angiotensinogen (UniProt ID A0A2I2UUP6), as measured by mass spectrometry, was compared with that measured by ELISA. The two methods showed a strong and significant correlation in both training (Spearman's 𝜌 = 0.84; *p*‐value < 0.001) and testing (Spearman's 𝜌 = 0.84; *p*‐value < 0.001) data (Figure 5), validating the relative protein quantification of our mass spectrometry protocol.

### 3.5 Performance of selected subset of proteins

Following the method described in Section 2.4, an elastic net regression model was developed. The optimal alpha identified was 0.1; 135 out of 266 proteins were retained in the model (Table S7). Using this smaller set of proteins, another random forest model was trained and tested following the same pipeline as described above. Under the ROC curve analysis, the model using this subset of proteins demonstrated an AUC of 0.977 (95% CI, 0.942–1.000).

## 4 DISCUSSION

We explored the use of mass spectrometry‐based urine proteomic analysis in cats with CKD and its potential use for CKD diagnosis. We characterized changes in the urinary proteome in cats with CKD. We further showed that the urinary proteome pattern can diagnose CKD in client‐owned cats with high sensitivity and specificity. Meanwhile, a single biomarker, retinol‐binding protein 4 (RBP4), might be able to achieve comparable diagnostic performance on its own. We also showed that a smaller panel identified by elastic net regression (135 proteins versus 266 proteins in the full panel) might be sufficient for CKD diagnosis.

Commonly referred to as retinol‐binding protein or serum retinol‐binding protein, RBP4 is a 21 kDa protein freely filtered through the glomerulus and usually reabsorbed in proximal tubules (Norden et al., 2014). Increased urinary RBP4 has been proposed as a marker of tubulointerstitial damage and impaired tubular reabsorption in humans (Bernard et al., 1987; Cutillas et al., 2004; Pallet et al., 2014) and dogs (Chacar et al., 2017; Nabity et al., 2012; Raila et al., 2010). Intriguingly, RBP4 has emerged as a promising biomarker for chronic kidney disease of uncertain etiology (CKDu; also known as Mesoamerican nephropathy) in human beings (Fernando et al., 2019; Kolli et al., 2023; Smpokou, 2021). This particular subtype of CKD is characterized by tubulointerstitial lesions (Weaver et al., 2015; Wijkström et al., 2017), which match the classic pathological findings in feline CKD (Brown et al., 2016). Previous studies in cats using immunoassay (van Hoek et al., 2008; Van Hoek et al., 2009) or gel‐based urinary proteomic analysis (Ferlizza et al., 2015) have also documented a notable increase in urinary RBP4 in cats with CKD. Further, urinary abundance of cubilin—a multiligand proximal tubular protein responsible for protein reabsorption—was significantly decreased in the CKD group compared to the healthy group. This has been noted as a unique finding in human CKDu patients (Kolli et al., 2023). Together, these results support pathophysiological similarity between CKD in cats and CKDu in humans. Findings in feline CKD might, therefore, provide valuable information about this disease in humans.

In terms of changes in individual proteins' urinary abundance during CKD, our results align with most previous studies using gel‐based quantification, including increased abundance of RBP4, cystatin M, albumin, and transferrin in urine from CKD cats (Crisi et al., 2020; Ferlizza et al., 2015). Meanwhile, we identified potential urinary biomarkers that have not been described in cats but have been documented in urine from other species, such as gelsolin (Ferreira et al., 2011) and inter‐alpha inhibitor (Hu et al., 2021; Jiang et al., 2022; Marengo et al., 1998).

The main significance of this study is the exploration and validation of mass spectrometry‐based urinary proteomic analysis in domestic cats. Our training and test sets represented a diverse sample of cats (i.e., purpose‐bred institution‐owned cats with surgically induced CKD, and client‐owned companion cats with naturally occurring CKD). We demonstrated that, despite the differences, changes in the urinary proteome during CKD are consistent, and the random forest model trained on institution‐owned cats with surgically induced CKD can accurately identify naturally occurring CKD. While the model yielded good diagnostic value, an almost equally impressive diagnostic value was found for RBP4. Further research is needed to determine whether a proteomic analysis is superior to urinary RBP4 as a diagnostic tool, especially given that the latter might be more cost‐efficient for clinical application.

Meanwhile, the difference in etiology of CKD between the training and test cohorts is also a major limitation of this study. The model only captures urinary protein signatures that were common between the surgically induced and naturally occurring CKD. In particular, if the “triggering event” of naturally occurring CKD has its distinct urinary protein signature, our study would not have captured such signatures.

While this study provides a proof of concept for urinary proteomics reflecting kidney injury, it could not compare the performance of urinary proteomic analysis with the current diagnostic standard (mainly based on serum creatinine) (IRIS Guidelines, 2023), which was used for CKD diagnosis in our test set (Lourenço et al., 2026). Serum creatinine is a late marker with multiple other limitations (Kongtasai et al., 2022), and a main pursuit in biomarker discovery is to find one that can either replace or enhance its performance. In our training set, where the CKD group was defined by partial nephrectomy (Huang et al., 2025; Schmiedt et al., 2023), the model diagnosed surgically induced CKD almost as precisely as serum creatinine concentration. It should be noted that in this cohort, while CKD was defined by partial nephrectomy instead of serum creatinine, the definition of the control group included a normal serum creatinine concentration (i.e., ≤1.8 mg/dL). Therefore, comparing the diagnostic ability between serum creatinine and our model might still favor serum creatinine unjustly, given that it was part of the grouping criteria.

Future prospective studies with long‐term follow‐up in at‐risk populations, similar to those conducted by Jepson and colleagues (Jepson et al., 2013), could help determine whether urine proteomic analysis can detect kidney injury sooner than serum creatinine concentration. Similarly, a prospective study in cats with CKD has the potential to determine whether urinary proteomic analysis predicts disease progression.

## AUTHOR CONTRIBUTIONS

**Jane H. C. Huang:** Conceptualization; data curation; formal analysis; funding acquisition; investigation; methodology; project administration; software; validation; visualization. **Bianca N. Lourenço:** Conceptualization; data curation; funding acquisition; project administration; resources; supervision. **Chad W. Schmiedt:** Resources. **Dennis R. Phillips:** Conceptualization; funding acquisition; methodology; validation.

## FUNDING INFORMATION

Morris Animal Foundation Feline Health pilot grant, grant number: D25FE‐830 and D24FE‐039 (to Dr. Bianca N Lourenço).

## CONFLICT OF INTEREST STATEMENT

Jane HC Huang and Dennis Phillips declare no conflicts of interest, financial or otherwise. Bianca Lourenço has received research funding from Ceva Animal Health, Boehringer Ingelheim Vetmedica GmbH, Porus GmBH, Dechra Veterinary Products, and Elanco Animal Health, and speaker honoraria from Boehringer Ingelheim Vetmedica, Ceva Animal Health, IDEXX Ltd., Antech Diagnostics, and Nestlé Purina. Chad Schmiedt has received research funding from Ceva Animal Health, Boehringer Ingelheim Vetmedica GmbH, Porus GmBH, Dechra Veterinary Products, and Elanco Animal Health. None of the commercial entities listed above had any role or influence on the study design, data collection, data analysis, results interpretation, manuscript preparation, or conclusions.

## ETHICS STATEMENT

All experimental procedures were approved by the University of Georgia Institutional Animal Care and Use Committee (protocol No. A2018 10‐001 and A2025 07‐019) and the Clinical Research Committee of the University of Georgia College of Veterinary Medicine (approval No. CR‐665).

## Supporting information


Appendix S1.

## Data Availability

The mass spectrometry proteomics data have been deposited to the ProteomeXchange Consortium via the PRIDE (Perez‐Riverol et al., 2025) partner repository with the dataset identifier PXD076696.
