# Supplementary material for: Mass spectrometry‐based urinary proteomic analysis in feline chronic kidney disease
Source: Physiol Rep. 2026 Aug 21;14(16):e71072. doi: 10.14814/phy2.71072 (PMC13498864; doi:10.14814/phy2.71072)
Supplement: Supplementary file 1 [file PHY2-14-e71072-s001.docx]

**Supplemental Figure S1**. Principal component analysis (PCA) of urine collected from parent study #1 (1). Each dot represents one cat. The original study included 15 institution-owned cats with surgically induced CKD and 15 community-owned healthy cats. For the current study, 1 CKD cat deceased before urine sample collection, and 1 healthy cat did not have a sufficient urine sample, resulting in 14 cats in the CKD group (green dots; 8 spayed females, 6 castrated males) and 14 cats in the control group (blue dots; 8 spayed females, 6 castrated males). Analysis revealed major allergen I polypeptide chain 1 (UniProt ID P30438) and hemoglobin subunits (UniProt ID P07412 and P07405) being major drivers for samples clustering, and a confounding effect caused by sample collection methods (voided urine collected from litter box in CKD group; cystocentesis in control group) was suspected. CKD, chronic kidney disease; Cysto, cystocentesis; PC, principal component.

**Supplemental Figure S2**. Receiver operating characteristic (ROC) curve analysis in the training set (n= 38; 14 cats with surgically-induced CKD, 24 healthy controls). Analysis was conducted using R package pROC (version 1.19.0.1); shaded regions around each curve represent the 95% confidence interval computed using DeLong's method. (A) ROC curve for the urinary proteomic random forest model-predicted CKD probability on out-of-bag samples from the training set, provided by R package caret (version 7.0.1) (2) (AUC= 0.969; 95% CI 0.924–1.000; pink). (B) ROC curves for two conventional clinical renal biomarkers: serum creatinine (AUC= 0.984; 95% CI 0.956–1.000; pink) and urine protein-to-creatinine ratio (UPC; AUC = 0.498; 95% CI 0.285–0.712; sage).

1.	Huang JH, Lourenço BN, Schmiedt CW, Coleman AE. Classical and alternative renin-angiotensin-aldosterone systems in a feline remnant kidney model. American Journal of Veterinary Research. 2025;86(11).

2.	Kuhn M. Building Predictive Models in R Using the caret Package. Journal of Statistical Software. 2008;28(5):1 - 26.

The original Excel files for supplemental tables (tables S1-S7) can be found in the following repository:

<https://figshare.com/s/1684f6da06f26db64a16>

Provided below is information copied from the Excel files.

**Supplemental Table S1.** Details on training cohort.

| **ID** | **Source** | **Group** | **Sex** | **Breed** | **Age, year** | **Serum Creatinine (mg/dL)** | **Blood Urea Nitrogen (mg/dL)** | **Serum SDMA (𝜇g/dL)** | **Urine-Specific Gravity** | **Urinary Protein (mg/dL)** | **Urinary Creatinine (mg/dL)** | **UPC** |
| --- | --- | --- | --- | --- | --- | --- | --- | --- | --- | --- | --- | --- |
| cat #1 | Community-owned | Control, Cystocentasis | Male, Castrated | Domestic Short Hair | 3.48 | 1.7 | 26 | 14 | 1.057 | 63 | 545.9 | 0.12 |
| cat #2 | Community-owned | Control, Cystocentasis | Male, Castrated | Domestic Median Hair | 3.25 | 1.3 | 19 | 14 | 1.046 | 46 | 359.9 | 0.13 |
| cat #3 | Community-owned | Control, Cystocentasis | Male, Castrated | Siamese | 2.43 | 1.7 | 23 | 11 | 1.046 | 79 | 257.7 | 0.31 |
| cat #4 | Community-owned | Control, Cystocentasis | Male, Castrated | Siamese | 2.43 | 1.5 | 23 | 9 | 1.052 | 44 | 408.1 | 0.11 |
| cat #5 | Community-owned | Control, Cystocentasis | Male, Castrated | Domestic Median Hair | 2.27 | 1.5 | 22 | 12 | 1.035 | 29 | 306.8 | 0.09 |
| cat #6 | Community-owned | Control, Cystocentasis | Male, Castrated | Domestic Short Hair | 2.41 | 1.2 | 21 | 11 | 1.053 | 26 | 289.9 | 0.09 |
| cat #7 | Community-owned | Control, Cystocentasis | Female, Spayed | Domestic Short Hair | 2.31 | 1.2 | 27 | 13 | 1.053 | 38 | 270 | 0.14 |
| cat #8 | Community-owned | Control, Cystocentasis | Female, Spayed | Domestic Short Hair | 3.42 | 1.8 | 29 | 13 | 1.051 | 30 | 359.7 | 0.08 |
| cat #9 | Community-owned | Control, Cystocentasis | Female, Spayed | Sphynx | 3.55 | 0.6 | 17 | 8 | 1.053 | 29 | 170.6 | 0.17 |
| cat #10 | Community-owned | Control, Cystocentasis | Female, Spayed | Sphynx | 3.55 | 0.5 | 17 | 7 | 1.056 | 35 | 156.8 | 0.22 |
| cat #11 | Community-owned | Control, Cystocentasis | Female, Spayed | Domestic Short Hair | 3.04 | 1.5 | 20 | 10 | 1.045 | 23 | 304.1 | 0.08 |
| cat #12 | Community-owned | Control, Cystocentasis | Female, Spayed | Domestic Short Hair | 4 | 1.4 | 26 | 12 | 1.028 | 19 | 204.4 | 0.09 |
| cat #13 | Community-owned | Control, Cystocentasis | Female, Spayed | Domestic Short Hair | 3.83 | 1.6 | 22 | 10 | 1.041 | 32 | 332.4 | 0.1 |
| cat #14 | Community-owned | Control, Cystocentasis | Female, Spayed | Domestic Short Hair | 4.44 | 1.2 | 26 | 12 | 1.055 | 64 | 327.9 | 0.2 |
| cat #15 | Institution-owned | CKD | Male, Castrated | Domestic Short Hair | 4.9 | 1.6 | 21 | Not measured | 1.021 | 8 | 121.4 | 0.07 |
| cat #16 | Institution-owned | CKD | Male, Castrated | Domestic Short Hair | 5.06 | 2 | 22 | Not measured | 1.035 | 38 | 266.1 | 0.14 |
| cat #17 | Institution-owned | CKD | Male, Castrated | Domestic Short Hair | 5.02 | 1.9 | 22 | Not measured | 1.043 | 31 | 303.8 | 0.1 |
| cat #18 | Institution-owned | CKD | Male, Castrated | Domestic Short Hair | 4.83 | 2.1 | 20 | Not measured | 1.047 | 40 | 379 | 0.11 |
| cat #19 | Institution-owned | CKD | Male, Castrated | Domestic Short Hair | 5.07 | 2.2 | 23 | Not measured | 1.047 | 41 | 313 | 0.13 |
| cat #20 | Institution-owned | CKD | Male, Castrated | Domestic Short Hair | 5.05 | 2.5 | 27 | Not measured | 1.014 | 24 | 64.9 | 0.37 |
| cat #21 | Institution-owned | CKD | Female, Spayed | Domestic Short Hair | 5.11 | 1.8 | 17 | Not measured | 1.024 | 8 | 169.8 | 0.05 |
| cat #22 | Institution-owned | CKD | Female, Spayed | Domestic Short Hair | 5.1 | 1.8 | 22 | Not measured | 1.018 | 13 | 89.7 | 0.14 |
| cat #23 | Institution-owned | CKD | Female, Spayed | Domestic Short Hair | 5.11 | 2 | 20 | Not measured | >1.060 | 92 | 675.1 | 0.14 |
| cat #24 | Institution-owned | CKD | Female, Spayed | Domestic Short Hair | 5.08 | 2 | 22 | Not measured | 1.041 | 21 | 282.8 | 0.07 |
| cat #25 | Institution-owned | CKD | Female, Spayed | Domestic Short Hair | 5.08 | 4 | 37 | Not measured | 1.025 | 16 | 192.8 | 0.08 |
| cat #26 | Institution-owned | CKD | Female, Spayed | Domestic Short Hair | 5.08 | 2.8 | 26 | Not measured | 1.018 | 28 | 104.6 | 0.27 |
| cat #27 | Institution-owned | CKD | Female, Spayed | Domestic Short Hair | 5.1 | 2.4 | 24 | Not measured | 1.031 | 15 | 232.4 | 0.06 |
| cat #28 | Institution-owned | CKD | Female, Spayed | Domestic Short Hair | 5.1 | 2.2 | 26 | Not measured | 1.037 | 23 | 212.2 | 0.11 |
| Cat #29 | Institution-owned | Control, Free-Catch | Female, Spayed | Domestic Short Hair | 2.83 | 1.4 | 24 | 16 | 1.054 | 25 | 341.4 | 0.07 |
| Cat #30 | Institution-owned | Control, Free-Catch | Female, Spayed | Domestic Short Hair | 2.92 | 1.2 | 27 | 14 | 1.054 | 41 | 354.8 | 0.12 |
| Cat #31 | Institution-owned | Control, Free-Catch | Female, Spayed | Domestic Short Hair | 2.93 | 1.4 | 25 | 14 | 1.058 | 34 | 447.9 | 0.08 |
| Cat #32 | Institution-owned | Control, Free-Catch | Female, Spayed | Domestic Short Hair | 2.81 | 1.1 | 18 | 12 | >1.060 | 24 | 408.9 | 0.06 |
| Cat #33 | Institution-owned | Control, Free-Catch | Female, Spayed | Domestic Short Hair | 2.81 | 1.1 | 23 | 13 | >1.060 | 28 | 377.8 | 0.07 |
| Cat #34 | Institution-owned | Control, Free-Catch | Male, Castrated | Domestic Short Hair | 2.85 | 1.4 | 19 | 14 | 1.048 | 44 | 382.9 | 0.11 |
| Cat #35 | Institution-owned | Control, Free-Catch | Male, Castrated | Domestic Short Hair | 2.92 | 1.2 | 16 | 13 | 1.058 | 52 | 518.7 | 0.1 |
| Cat #36 | Institution-owned | Control, Free-Catch | Male, Castrated | Domestic Short Hair | 2.85 | 1.4 | 18 | 11 | >1.060 | 64 | 474.3 | 0.13 |
| Cat #37 | Institution-owned | Control, Free-Catch | Male, Castrated | Domestic Short Hair | 2.88 | 1.6 | 21 | 13 | >1.060 | 56 | 591.1 | 0.09 |

**Supplemental Table S2.** Logistic regression analysis for group CKD versus control.

| **Urinary protein** | **UniProt ID** | **Unadjust p-value** | **Adjusted p-value** | **Fold change** | **Odds ratio (95% CI)** |
| --- | --- | --- | --- | --- | --- |
| Cubilin | M3WJ25 | 0.001 | 0.049 | 0.35 | 0.127 (0.036-0.453) |
| ADF-H domain-containing protein | A0A5F5XRM0 | 0.002 | 0.049 | 3.378 | 9.28 (2.336-36.871) |
| CXADR Ig-like cell adhesion molecule | A0A337S2K2 | 0.002 | 0.049 | 2.785 | 4.643 (1.792-12.028) |
| Vascular cell adhesion molecule 1 | M3WED4 | 0.002 | 0.049 | 2.8 | 4.899 (1.788-13.425) |
| Fatty acid-binding protein, intestinal | M3WQY4 | 0.002 | 0.049 | 2.855 | 4.744 (1.748-12.873) |
| IaI heavy chain 4 | A0A5F5Y7V2 | 0.002 | 0.049 | 2.508 | 6.24 (1.921-20.275) |
| Protein C receptor | M3VYA2 | 0.002 | 0.049 | 0.4 | 0.204 (0.073-0.571) |
| NHL repeat containing 3 | A0A2I2UPA5 | 0.003 | 0.049 | 0.37 | 0.198 (0.069-0.568) |
| Lipase | A0A5F5XPV2 | 0.003 | 0.049 | 0.405 | 0.104 (0.024-0.463) |
| Alpha-amylase | M3X5H0 | 0.003 | 0.049 | 2.799 | 7.631 (2-29.117) |
| Thy-1 membrane glycoprotein | A0A2I2UGV7 | 0.003 | 0.049 | 2.942 | 4.508 (1.669-12.174) |
| Gelsolin | A0A5F5XIG6 | 0.003 | 0.049 | 2.706 | 7.514 (1.976-28.576) |
| LDL receptor related protein 2 | M3W5B5 | 0.003 | 0.049 | 0.393 | 0.175 (0.054-0.56) |
| Actin, cytoplasmic 2 | M3W6I2 | 0.003 | 0.049 | 0.429 | 0.14 (0.038-0.522) |
| Cadherin 11 | M3W046 | 0.004 | 0.049 | 2.416 | 3.131 (1.455-6.736) |
| N-acylethanolamine-hydrolyzing acid amidase | A0A2I2V455 | 0.004 | 0.049 | 0.412 | 0.116 (0.027-0.494) |
| Aminopeptidase N | A0A337SS27 | 0.004 | 0.049 | 0.434 | 0.245 (0.095-0.631) |
| Secreted phosphoprotein 1 | A0A2I2UVQ2 | 0.004 | 0.049 | 2.563 | 3.785 (1.542-9.29) |
| Gamma-interferon-inducible lysosomal thiol reductase | M3WSA1 | 0.004 | 0.049 | 0.417 | 0.309 (0.139-0.684) |
| Desmocollin 2 | A0A5F5XHZ3 | 0.004 | 0.049 | 2.411 | 4.799 (1.645-14.002) |
| Cystatin E/M | M3WIK5 | 0.004 | 0.049 | 2.62 | 41.584 (3.216-537.738) |
| Metalloendopeptidase | M3WB64 | 0.004 | 0.049 | 2.396 | 3.26 (1.447-7.343) |
| Filamin A | A0A2I2V3V8 | 0.005 | 0.049 | 2.442 | 3.204 (1.43-7.18) |
| Cadherin 13 | M3W235 | 0.005 | 0.049 | 2.342 | 9.18 (1.971-42.742) |
| Cadherin-1 | A0A337SG59 | 0.005 | 0.049 | 2.19 | 5.459 (1.679-17.744) |
| Integral membrane protein 2 | A0A2I2UVC1 | 0.005 | 0.049 | 0.393 | 0.199 (0.065-0.613) |
| Desmocollin 1 | A0A2I2UZT4 | 0.005 | 0.049 | 2.417 | 9.961 (2-49.621) |
| Prosaposin | M3WI47 | 0.005 | 0.049 | 0.45 | 0.172 (0.05-0.591) |
| Plasminogen | M3X3T9 | 0.005 | 0.049 | 2.236 | 5.027 (1.613-15.67) |
| Retinol binding protein 4 | M5AXY1 | 0.006 | 0.049 | 2.329 | 3.75 (1.471-9.558) |
| Serpin family A member 1 | A0A5F5XW04 | 0.006 | 0.049 | 2.043 | 3.695 (1.456-9.379) |
| Afamin | A0A2I2U1Z3 | 0.006 | 0.049 | 2.325 | 2.611 (1.312-5.197) |
| CD44 antigen | A0A337SMK2 | 0.007 | 0.049 | 0.477 | 0.22 (0.073-0.66) |
| Insulin-like growth factor-binding protein 1 | M3W4L2 | 0.007 | 0.049 | 0.416 | 0.271 (0.105-0.701) |
| Immunoglobulin superfamily containing leucine rich repeat | M3X315 | 0.007 | 0.049 | 0.459 | 0.317 (0.137-0.732) |
| Transferrin | M3X260 | 0.007 | 0.049 | 2.301 | 3.178 (1.364-7.407) |
| MBL associated serine protease 2 | A0A5F5Y096 | 0.007 | 0.049 | 0.301 | 0.034 (0.003-0.405) |
| Nucleobindin-1 | M3W064 | 0.008 | 0.049 | 0.489 | 0.289 (0.116-0.721) |
| Urokinase-type plasminogen activator | A0A337S474 | 0.008 | 0.049 | 0.452 | 0.068 (0.009-0.494) |
| Immunoglobulin superfamily member 8 | A0A2I2U7U8 | 0.008 | 0.049 | 2.208 | 2.653 (1.29-5.459) |
| Matrix remodeling-associated protein 8 | A0A337SH59 | 0.008 | 0.049 | 0.479 | 0.285 (0.112-0.721) |
| Albumin | P49064 | 0.008 | 0.049 | 2.288 | 2.889 (1.317-6.337) |
| receptor protein-tyrosine kinase | A0A2I2UTQ4 | 0.008 | 0.049 | 0.486 | 0.337 (0.151-0.755) |
| Sushi domain-containing protein | A0A337S3R0 | 0.008 | 0.049 | 0.489 | 0.311 (0.131-0.741) |
| Transmembrane protein 277 | A0A5F5Y3H5 | 0.009 | 0.049 | 0.495 | 0.266 (0.099-0.713) |
| Sulfotransferase | M3WQA9 | 0.009 | 0.049 | 0.45 | 0.331 (0.145-0.755) |
| Antithrombin-III | M3WLL8 | 0.009 | 0.049 | 2.185 | 3.016 (1.319-6.895) |
| LY6/PLAUR domain containing 2 | M3VU52 | 0.009 | 0.05 | 2.131 | 3.123 (1.327-7.352) |

**Supplemental Table S3.** Linear regression analysis versus serum creatinine.

| **Urinary protein** | **UniProt ID** | **Unadjusted p-value** | **Adjusted p-value** | **Estimate (95% CI)** |
| --- | --- | --- | --- | --- |
| Cystatin C | D5MTH1 | <0.001 | 0.001 | 0.43 [0.27, 0.58] |
| ADF-H domain-containing protein | A0A5F5XRM0 | <0.001 | 0.003 | 0.31 [0.17, 0.45] |
| IaI heavy chain 4 | A0A5F5Y7V2 | <0.001 | 0.003 | 0.36 [0.2, 0.52] |
| LDL receptor related protein 2 | M3W5B5 | <0.001 | 0.003 | -0.35 [-0.51, -0.2] |
| Cystatin E/M | M3WIK5 | <0.001 | 0.003 | 0.37 [0.22, 0.53] |
| Retinol binding protein 4 | M5AXY1 | <0.001 | 0.003 | 0.34 [0.2, 0.49] |
| Cystatin domain-containing protein | A0A337SE08 | <0.001 | 0.004 | 0.36 [0.19, 0.52] |
| Gelsolin | A0A5F5XIG6 | <0.001 | 0.004 | 0.33 [0.18, 0.49] |
| Alpha-amylase | M3X5H0 | <0.001 | 0.004 | 0.33 [0.17, 0.49] |
| Prosaposin | M3WI47 | <0.001 | 0.009 | -0.34 [-0.52, -0.16] |
| Nidogen 2 | A0A337SMW2 | 0.001 | 0.009 | 0.31 [0.14, 0.48] |
| Lipase | A0A5F5XPV2 | 0.001 | 0.009 | -0.34 [-0.52, -0.15] |
| Adiposis regulatory factor | M3W8Q3 | 0.001 | 0.009 | 0.33 [0.15, 0.51] |
| Transmembrane protein 277 | A0A5F5Y3H5 | 0.001 | 0.012 | -0.33 [-0.51, -0.15] |
| N-acylethanolamine-hydrolyzing acid amidase | A0A2I2V455 | 0.001 | 0.014 | -0.32 [-0.5, -0.14] |
| Kinesin-like protein | A0A5F5XE91 | 0.001 | 0.014 | 0.32 [0.14, 0.51] |
| Urokinase-type plasminogen activator | A0A337S474 | 0.001 | 0.015 | -0.31 [-0.5, -0.13] |
| Proenkephalin-A | A0A5F5XWQ5 | 0.001 | 0.015 | 0.31 [0.13, 0.49] |
| CD44 antigen | A0A337SMK2 | 0.002 | 0.017 | -0.31 [-0.5, -0.13] |
| Cadherin 13 | M3W235 | 0.002 | 0.017 | 0.3 [0.12, 0.49] |

**Supplemental Table S4.** Details on test cohort.

| **ID** | **Srouce** | **Group** | **Sex** | **Breed** | **Age, year** | **Serum Creatinine (mg/dL)** | **Blood Urea Nitrogen (mg/dL)** | **Serum SDMA (𝜇g/dL)** | **Urine-Specific Gravity** | **Urinary Protein (mg/dL)** | **Urinary Creatinine (mg/dL)** | **UPC** | **Model-predicted CKD probability** |
| --- | --- | --- | --- | --- | --- | --- | --- | --- | --- | --- | --- | --- | --- |
| Cat26 | Client-owned | Control | Female, spayed | Domestic short hair | 5.1 | 0.7 | 27 | 7 | >1.060 | 39 | 289.8 | 0.13 | 9.40% |
| Cat32 | Client-owned | Control | Female, spayed | Domestic short hair | 12 | 1.2 | 25 | 9 | 1.044 | 29 | 252.9 | 0.11 | 16.60% |
| Cat39 | Client-owned | Control | Female, spayed | Domestic long hair | 6.1 | 1.6 | 21 | 12 | 1.053 | 27 | 365.9 | 0.07 | 19.20% |
| Cat28 | Client-owned | Control | Female, spayed | Domestic short hair | 10.5 | 1 | 22 | 11 | 1.045 | 26 | 193.3 | 0.13 | 20.40% |
| Cat3 | Client-owned | Control | Female, spayed | Domestic short hair | 12.2 | 1 | 29 | 9 | >1.060 | 55 | 344.5 | 0.16 | 21.40% |
| Cat2 | Client-owned | Control | Female, spayed | Bengal | 11.5 | 0.9 | 23 | 11 | 1.057 | 38 | 275.4 | 0.14 | 24.60% |
| Cat40 | Client-owned | Control | Male, castrated | Domestic short hair | 9.6 | 1.2 | 22 | 11 | 1.054 | 57 | 366.5 | 0.16 | 26.00% |
| Cat37 | Client-owned | Control | Female, spayed | Domestic short hair | 14.1 | 1.2 | 22 | 8 | 1.048 | 31 | 288.1 | 0.11 | 26.20% |
| Cat8 | Client-owned | Control | Male, castrated | Domestic long hair | 11.6 | 1.1 | 23 | 8 | >1.060 | 24 | 225 | 0.11 | 26.60% |
| Cat9 | Client-owned | Control | Male, castrated | Domestic median hair | 8.2 | 1.4 | 25 | 9 | 1.048 | 39 | 304.8 | 0.13 | 26.60% |
| Cat21 | Client-owned | Control | Female, spayed | Domestic median hair | 9.8 | 1.1 | 17 | 8 | 1.051 | 30 | 312.4 | 0.1 | 29.80% |
| Cat38 | Client-owned | Control | Male, castrated | Domestic short hair | 9.8 | 1.3 | 22 | 14 | 1.034 | 24 | 257.3 | 0.09 | 36.00% |
| Cat35 | Client-owned | Control | Male, castrated | Ragdoll | 14.9 | 1.1 | 22 | 11 | 1.056 | 39 | 319.4 | 0.12 | 43.00% |
| Cat25 | Client-owned | CKD | Female, spayed | Siamese | 10.7 | 1.9 | 64 | 15 | 1.019 | 4 | 57.2 | 0.07 | 44.00% |
| Cat24 | Client-owned | Control | Male, castrated | Domestic short hair | 12.1 | 1.5 | 29 | 12 | 1.057 | 61 | 437.6 | 0.14 | 46.80% |
| Cat10 | Client-owned | CKD | Male, castrated | Domestic long hair | 14 | 2.1 | 28 | 15 | 1.019 | 17 | 104.3 | 0.16 | 49.00% |
| Cat17 | Client-owned | Control | Male, castrated | Domestic median hair | 15.1 | 1.5 | 24 | 13 | 1.048 | 50 | 310.3 | 0.16 | 49.60% |
| Cat6 | Client-owned | Control | Female, spayed | Domestic short hair | 17.1 | 0.9 | 23 | 9 | 1.03 | 39 | 146.5 | 0.27 | 51.00% |
| Cat11 | Client-owned | Control | Female, spayed | Domestic short hair | 14.2 | 0.9 | 21 | 11 | 1.053 | 54 | 238.8 | 0.23 | 54.60% |
| Cat20 | Client-owned | CKD | Female, spayed | Domestic short hair | 13 | 1.6 | 33 | 13 | 1.019 | 9 | 97.3 | 0.09 | 54.80% |
| Cat16 | Client-owned | CKD | Female, spayed | Domestic short hair | 13.4 | 2.9 | 37 | 18 | 1.014 | 37 | 83 | 0.45 | 55.60% |
| Cat19 | Client-owned | CKD | Male, castrated | Domestic short hair | 17.9 | 2.2 | 29 | 27 | 1.016 | 12 | 122.2 | 0.1 | 57.00% |
| Cat33 | Client-owned | CKD | Female, spayed | Domestic short hair | 17.1 | 2.2 | 37 | 20 | 1.015 | 12 | 96.1 | 0.12 | 57.60% |
| Cat18 | Client-owned | CKD | Male, castrated | Domestic short hair | 17.5 | 2.3 | 34 | 24 | 1.025 | 41 | 170.3 | 0.24 | 58.60% |
| Cat27 | Client-owned | CKD | Male, castrated | Domestic short hair | 13.4 | 1.9 | 29 | 15 | 1.026 | 27 | 213.9 | 0.13 | 62.80% |
| Cat30 | Client-owned | CKD | Female, spayed | Domestic short hair | 18.1 | 2.1 | 49 | 10 | 1.017 | 12 | 66.9 | 0.18 | 63.40% |
| Cat13 | Client-owned | CKD | Male, castrated | Domestic long hair | 15.8 | 1.7 | 39 | 18 | 1.029 | 59 | 126.8 | 0.47 | 68.40% |
| Cat36 | Client-owned | CKD | Female, spayed | Domestic short hair | 5.3 | 2.8 | 39 | 16 | 1.029 | 118 | 276.2 | 0.43 | 69.60% |
| Cat12 | Client-owned | CKD | Male, castrated | Domestic short hair | 10.6 | 2.1 | 45 | 22 | 1.024 | 18 | 138.2 | 0.13 | 71.40% |
| Cat5 | Client-owned | CKD | Male, castrated | Domestic long hair | 16.7 | 2.7 | 51 | 22 | 1.017 | 8 | 73.8 | 0.11 | 72.00% |
| Cat14 | Client-owned | CKD | Female, spayed | Domestic short hair | 4.5 | 4.7 | 61 | 25 | 1.018 | 27 | 139.1 | 0.19 | 72.00% |
| Cat15 | Client-owned | CKD | Female, spayed | Domestic short hair | 15.1 | 1.6 | 33 | 20 | 1.015 | 14 | 61.4 | 0.23 | 72.40% |
| Cat1 | Client-owned | CKD | Male, castrated | Domestic short hair | 13.5 | 2 | 54 | 13 | 1.027 | 21 | 117.1 | 0.18 | 72.80% |
| Cat29 | Client-owned | CKD | Female, spayed | Domestic short hair | 14.3 | 2 | 43 | 26 | 1.023 | 34 | 107.5 | 0.32 | 75.40% |
| Cat7 | Client-owned | CKD | Female, spayed | Domestic short hair | 12.2 | 1.6 | 64 | 13 | 1.018 | 10 | 43.7 | 0.23 | 79.80% |
| Cat23 | Client-owned | CKD | Female, spayed | Domestic short hair | 12.6 | 2.8 | 33 | 20 | 1.03 | 30 | 262.3 | 0.11 | 82.40% |
| Cat22 | Client-owned | CKD | Male, castrated | Domestic long hair | 14.6 | 3.7 | 45 | 33 | 1.019 | 34 | 147 | 0.23 | 83.60% |
| Cat4 | Client-owned | CKD | Female, spayed | Domestic short hair | 19.2 | 1.8 | 37 | 16 | 1.015 | 22 | 70.9 | 0.31 | 86.20% |
| Cat31 | Client-owned | CKD | Female, spayed | Domestic long hair | 13.5 | 3.1 | 59 | 20 | 1.02 | 17 | 100.9 | 0.17 | 87.20% |
| Cat34 | Client-owned | CKD | Female, spayed | Domestic short hair | 13.5 | 2.5 | 44 | 16 | 1.014 | 22 | 66.5 | 0.33 | 92.00% |

**Supplemental Table S5.** Urinary proteins' overall importance for the prediction model.

| **Urinary protein** | **UniProt ID** | **Overall Importance** | **Direction** |
| --- | --- | --- | --- |
| MBL associated serine protease 2 | A0A5F5Y096 | 3.227 | Lower in CKD |
| Cystatin E/M | M3WIK5 | 1.381 | Higher in CKD |
| ADF-H domain-containing protein | A0A5F5XRM0 | 1.154 | Higher in CKD |
| Prosaposin | M3WI47 | 0.893 | Lower in CKD |
| Lipase | A0A5F5XPV2 | 0.812 | Lower in CKD |
| Actin, cytoplasmic 2 | M3W6I2 | 0.59 | Lower in CKD |
| Cubilin | M3WJ25 | 0.535 | Lower in CKD |
| Vascular cell adhesion molecule 1 | M3WED4 | 0.517 | Higher in CKD |
| N-acylethanolamine-hydrolyzing acid amidase | A0A2I2V455 | 0.434 | Lower in CKD |
| Protein C receptor | M3VYA2 | 0.417 | Lower in CKD |
| CD44 antigen | A0A337SMK2 | 0.274 | Lower in CKD |
| Gelsolin | A0A5F5XIG6 | 0.26 | Higher in CKD |
| CXADR Ig-like cell adhesion molecule | A0A337S2K2 | 0.256 | Higher in CKD |
| Peroxiredoxin-6 | A0A2I2UL25 | 0.255 | Higher in CKD |
| Stratifin | A0A5F5Y312 | 0.228 | Higher in CKD |
| Secreted phosphoprotein 1 | A0A2I2UVQ2 | 0.211 | Higher in CKD |
| Aminopeptidase N | A0A337SS27 | 0.207 | Lower in CKD |
| Apolipoprotein A-I | M3WPG6 | 0.192 | Higher in CKD |
| Urokinase-type plasminogen activator | A0A337S474 | 0.179 | Lower in CKD |
| Alpha-amylase | M3X5H0 | 0.177 | Higher in CKD |
| Filamin A | A0A2I2V3V8 | 0.17 | Higher in CKD |
| Cadherin 13 | M3W235 | 0.161 | Higher in CKD |
| Desmocollin 2 | A0A5F5XHZ3 | 0.161 | Higher in CKD |
| Thy-1 membrane glycoprotein | A0A2I2UGV7 | 0.148 | Higher in CKD |
| Afamin | A0A2I2U1Z3 | 0.137 | Higher in CKD |
| Corticosteroid-binding globulin | M3WCX0 | 0.137 | Lower in CKD |
| Desmocollin 1 | A0A2I2UZT4 | 0.113 | Higher in CKD |
| Napsin A aspartic peptidase | A0A5F5XWA2 | 0.112 | Lower in CKD |
| NHL repeat containing 3 | A0A2I2UPA5 | 0.111 | Lower in CKD |
| Renin receptor | M3XEW0 | 0.11 | Lower in CKD |
| LDL receptor related protein 2 | M3W5B5 | 0.105 | Lower in CKD |
| Proenkephalin-A | A0A5F5XWQ5 | 0.094 | Higher in CKD |
| Insulin like growth factor binding protein 7 | A0A2I2U3H1 | 0.092 | Lower in CKD |
| Hexosyltransferase | M3WAQ7 | 0.08 | Lower in CKD |
| Fatty acid-binding protein, intestinal | M3WQY4 | 0.079 | Higher in CKD |
| Integral membrane protein 2 | A0A2I2UVC1 | 0.074 | Lower in CKD |
| Cystatin domain-containing protein | A0A337SE08 | 0.073 | Higher in CKD |
| Ig-like domain-containing protein | A0A2I2U5M6 | 0.072 | Higher in CKD |
| Gamma-interferon-inducible lysosomal thiol reductase | M3WSA1 | 0.071 | Lower in CKD |
| Plasminogen | M3X3T9 | 0.068 | Higher in CKD |
| Immunoglobulin superfamily containing leucine rich repeat | M3X315 | 0.063 | Lower in CKD |
| Chromogranin-A | A0A5F5XNS6 | 0.063 | Higher in CKD |
| IaI heavy chain 4 | A0A5F5Y7V2 | 0.061 | Higher in CKD |
| Kinesin-like protein | A0A5F5XE91 | 0.057 | Higher in CKD |
| Dipeptidyl peptidase 4 | Q9N2I7 | 0.055 | Lower in CKD |
| Fel d 7 allergen | E5D2Z5 | 0.055 | Higher in CKD |
| Dipeptidyl peptidase 7 | A0A5F5XVS8 | 0.052 | Higher in CKD |
| Nuclear transport factor 2 | M3X0F5 | 0.045 | Lower in CKD |
| LIM and SH3 domain protein 1 | M3X768 | 0.045 | Higher in CKD |
| receptor protein-tyrosine kinase | A0A2I2UTQ4 | 0.044 | Lower in CKD |
| LY6/PLAUR domain containing 2 | M3VU52 | 0.044 | Lower in CKD |
| Major allergen I polypeptide chain 1 | P30438 | 0.043 | Higher in CKD |
| Heparan sulfate proteoglycan 2 | A0A5F5Y6R8 | 0.043 | Higher in CKD |
| Cystatin C | D5MTH1 | 0.041 | Higher in CKD |
| Pentraxin family member | A0A5F5XWB9 | 0.038 | Lower in CKD |
| Transferrin | M3X260 | 0.037 | Higher in CKD |
| Cathepsin D | M3W8G3 | 0.037 | Lower in CKD |
| Solute carrier family 51 subunit beta | M3WSF5 | 0.036 | Lower in CKD |
| Fibrillin 1 | M3WMG2 | 0.035 | Higher in CKD |
| Actin, alpha skeletal muscle | M3XC93 | 0.034 | Lower in CKD |
| PDZ domain containing 1 | A0A2I2UBZ5 | 0.034 | Lower in CKD |
| Collectin-12 | M3WN82 | 0.031 | Higher in CKD |
| Adiposis regulatory factor | M3W8Q3 | 0.031 | Higher in CKD |
| Collagen type XVIII alpha 1 chain | A0A5F5XC75 | 0.028 | Lower in CKD |
| Cadherin-1 | A0A337SG59 | 0.028 | Higher in CKD |
| Lipocalin/cytosolic fatty-acid binding domain-containing protein | M3WIS7 | 0.028 | Higher in CKD |
| Tripeptidyl-peptidase 1 | A0A2I2V5I2 | 0.027 | Lower in CKD |
| Prothrombin | M3WSI8 | 0.024 | Higher in CKD |
| Ganglioside GM2 activator | M3WHT1 | 0.023 | Higher in CKD |
| Cadherin 11 | M3W046 | 0.023 | Higher in CKD |
| Immunoglobulin superfamily member 8 | A0A2I2U7U8 | 0.022 | Higher in CKD |
| Ezrin | M3W8S0 | 0.021 | Lower in CKD |
| Ig-like domain-containing protein | A0A5F5XZ27 | 0.021 | Higher in CKD |
| Insulin-like growth factor-binding protein 1 | M3W4L2 | 0.021 | Lower in CKD |
| Carboxylesterase 5A | Q8I034 | 0.021 | Lower in CKD |
| Trans-1,2-dihydrobenzene-1,2-diol dehydrogenase | A0A337S785 | 0.021 | Lower in CKD |
| Butyrophilin like 9 | A0A5F5XEY9 | 0.021 | Lower in CKD |
| Nidogen 1 | M3WED3 | 0.021 | Higher in CKD |
| Aminopeptidase | M3VU18 | 0.021 | Lower in CKD |
| Ig-like domain-containing protein | A0A5F5XP19 | 0.02 | Higher in CKD |
| Dimethylarginine dimethylaminohydrolase 2 | M3XC02 | 0.02 | Higher in CKD |
| Transmembrane protein 277 | A0A5F5Y3H5 | 0.019 | Lower in CKD |
| Metalloendopeptidase | M3WB64 | 0.019 | Higher in CKD |
| Lymphocyte antigen 6 family member D | A0A337SJ42 | 0.019 | Higher in CKD |
| Lactotransferrin | M3WKU0 | 0.019 | Higher in CKD |
| Fibrinogen alpha chain | M3W022 | 0.018 | Higher in CKD |
| Ig-like domain-containing protein | M3X4T8 | 0.018 | Higher in CKD |
| Restriction factor for feline retrovirus X | T2HUV9 | 0.018 | Higher in CKD |
| Junctional adhesion molecule A | Q2WGK2 | 0.018 | Higher in CKD |
| Fibulin-5 | M3WFS9 | 0.017 | Higher in CKD |
| Hemicentin 1 | M3X1M2 | 0.017 | Higher in CKD |
| Lectin, mannose binding 2 | M3XF94 | 0.016 | Higher in CKD |
| Pepsin A | M3WJA1 | 0.015 | Lower in CKD |
| Alkaline phosphatase, tissue-nonspecific isozyme | Q29486 | 0.015 | Lower in CKD |
| Endogenous retrovirus group V member 2 Env polyprotein-like | M3XBS7 | 0.015 | Lower in CKD |
| Secreted and transmembrane 1 | A0A5F5XU81 | 0.015 | Higher in CKD |
| Cartilage intermediate layer protein 2 | M3WKI3 | 0.014 | Higher in CKD |
| Sulfurtransferase | A0A337S0Y0 | 0.014 | Higher in CKD |
| Glutaminyl-peptide cyclotransferase | M3WST2 | 0.014 | Lower in CKD |
| Chymotrypsin C | M3W019 | 0.014 | Higher in CKD |
| Protein shisa-5 | A0A2I2UZT1 | 0.014 | Lower in CKD |
| N-sulfoglucosamine sulfohydrolase | M3VVP5 | 0.014 | Lower in CKD |
| Procollagen C-endopeptidase enhancer | A0A5F5XK05 | 0.014 | Higher in CKD |
| Hemoglobin subunit beta-A/B | P07412 | 0.014 | Higher in CKD |
| Peptidase inhibitor 16 | M3WXF2 | 0.014 | Higher in CKD |
| Glutathione peroxidase | A0A2I2V5E5 | 0.014 | Higher in CKD |
| Deoxyribonuclease-2-alpha | M3VUU5 | 0.014 | Lower in CKD |
| Haptoglobin | E3UTY9 | 0.014 | Higher in CKD |
| Conserved oligomeric Golgi complex subunit 4 | M3WBJ4 | 0.013 | Lower in CKD |
| Fibrillin 2 | M3WBW4 | 0.012 | Lower in CKD |
| Dermatopontin | M3XE34 | 0.012 | Higher in CKD |
| Dystroglycan 1 | A0A337SFQ4 | 0.011 | Higher in CKD |
| Uromodulin | M3W230 | 0.011 | Lower in CKD |
| N-acetylglucosamine-6-sulfatase | M3VUR6 | 0.011 | Lower in CKD |
| Basigin | A0A5F5Y008 | 0.011 | Higher in CKD |
| Ig-like domain-containing protein | A0A337SA21 | 0.011 | Higher in CKD |
| Collagen type I alpha 1 chain | M3W2F5 | 0.011 | Lower in CKD |
| Peptidase S1 domain-containing protein | M3X005 | 0.011 | Higher in CKD |
| Cell adhesion molecule 4 | A0A2I2ULF8 | 0.011 | Lower in CKD |
| Interleukin 1 receptor accessory protein | M3WNU0 | 0.011 | Lower in CKD |
| Biotinidase | A0A2I2U4L9 | 0.011 | Lower in CKD |
| Lipocalin/cytosolic fatty-acid binding domain-containing protein | A0A5F5XI37 | 0.011 | Lower in CKD |
| Anaphylatoxin-like domain-containing protein | A0A5F5XM97 | 0.011 | Higher in CKD |
| Prostaglandin-H2 D-isomerase | Q29487 | 0.011 | Higher in CKD |
| Sex hormone-binding globulin | A0A5F5XDP8 | 0.011 | Higher in CKD |
| MANSC domain containing 1 | A0A337SHI7 | 0.01 | Higher in CKD |
| Beta-2-microglobulin | Q5MGS7 | 0.01 | Higher in CKD |
| Ig-like domain-containing protein | A0A337S7W6 | 0.01 | Higher in CKD |
| ATPase H+ transporting accessory protein 1 | M3WF16 | 0.01 | Lower in CKD |
| EMI domain containing 1 | A0A2I2USC5 | 0.008 | Lower in CKD |
| Hemicentin 2 | M3X7U8 | 0.008 | Lower in CKD |
| Ribonuclease A family member k6 | M3WX61 | 0.008 | Higher in CKD |
| alpha-1,2-Mannosidase | A0A5F5Y426 | 0.008 | Lower in CKD |
| Proheparin-binding EGF-like growth factor | M3VWC1 | 0.008 | Lower in CKD |
| Arylsulfatase A | M3WDK2 | 0.008 | Lower in CKD |
| Collagen type VI alpha 3 chain | M3WHG4 | 0.008 | Lower in CKD |
| Vasorin | M3WEQ2 | 0.008 | Lower in CKD |
| Sulfhydryl oxidase | A0A5F5XIH0 | 0.008 | Higher in CKD |
| cathepsin X | M3VVI7 | 0.008 | Higher in CKD |
| Aquaporin-1 | A0A2I2UAM6 | 0.007 | Lower in CKD |
| Glycoprotein Ib platelet subunit alpha | A0A5F5XIK0 | 0.007 | Lower in CKD |
| Deoxyribonuclease | M3WGM9 | 0.007 | Lower in CKD |
| Superoxide dismutase [Cu-Zn] | A0A2I2UPH5 | 0.007 | Higher in CKD |
| Milk fat globule EGF and factor V/VIII domain containing | M3W678 | 0.007 | Lower in CKD |
| N-acetyl-alpha-glucosaminidase | M3WFH8 | 0.007 | Lower in CKD |
| Vitamin D-binding protein | M3W2Z1 | 0.007 | Higher in CKD |
| Insulin-like growth factor-binding protein 3 | A0A337S9R3 | 0.007 | Higher in CKD |
| Alpha-galactosidase | A0A2I2U1V5 | 0.007 | Lower in CKD |
| Lysosomal associated membrane protein 2 | A0A337STH0 | 0.007 | Lower in CKD |
| Antithrombin-III | M3WLL8 | 0.007 | Higher in CKD |
| Tenascin C | M3W358 | 0.007 | Lower in CKD |
| Phospholipase B-like | A0A2I2UDS4 | 0.007 | Lower in CKD |
| Sushi domain-containing protein | A0A337S3R0 | 0.007 | Lower in CKD |
| Nucleobindin-1 | M3W064 | 0.007 | Lower in CKD |
| Cochlin | M3WFG2 | 0.007 | Higher in CKD |
| Joining chain of multimeric IgA and IgM | M3XBL5 | 0.007 | Lower in CKD |
| Glyceraldehyde-3-phosphate dehydrogenase | A0A0A0MPZ5 | 0.007 | Higher in CKD |
| Serine protease 8 | A0A5F5XKF7 | 0.007 | Lower in CKD |
| Clusterin | M3WKP2 | 0.007 | Higher in CKD |
| Alpha-1-B glycoprotein | M3W0W4 | 0.007 | Lower in CKD |
| Serpin family D member 1 | M3W543 | 0.007 | Higher in CKD |
| Carboxylic ester hydrolase | M3W105 | 0.007 | Lower in CKD |
| Fibronectin | A0A2I2U011 | 0.006 | Lower in CKD |
| Major allergen I polypeptide chain 2 | P30440 | 0.004 | Higher in CKD |
| Angiotensin-converting enzyme | A0A2I2UTZ7 | 0.004 | Lower in CKD |
| Sulfotransferase | M3WQA9 | 0.004 | Lower in CKD |
| Collagen type I alpha 2 chain | M3WVN3 | 0.004 | Higher in CKD |
| Cadherin 3 | A0A2I2UB75 | 0.004 | Lower in CKD |
| Beta-glucuronidase | O97524 | 0.004 | Higher in CKD |
| Corneodesmosin | M3X143 | 0.004 | Higher in CKD |
| SH3 domain-binding glutamic acid-rich-like protein 3 | M3WHM8 | 0.004 | Lower in CKD |
| Cadherin related family member 5 | A0A5F5Y466 | 0.004 | Lower in CKD |
| Syndecan 4 | M3X6M9 | 0.004 | Lower in CKD |
| Heteroous nuclear ribonucleoprotein M | A0A5F5XQH4 | 0.004 | Lower in CKD |
| Albumin | P49064 | 0.004 | Higher in CKD |
| Lipocalin like 1 | M3XCK7 | 0.004 | Lower in CKD |
| Interleukin 18 binding protein | A0A2I2UF36 | 0.004 | Lower in CKD |
| Cathelicidin | F1C962 | 0.004 | Lower in CKD |
| Myelin protein zero like 2 | M3WFQ7 | 0.004 | Lower in CKD |
| Ig-like domain-containing protein | A0A2I2U3F6 | 0.004 | Higher in CKD |
| Basal cell adhesion molecule (Lutheran blood group) | M3W2U8 | 0.004 | Lower in CKD |
| Kininogen 1 | A0A5F5XVF5 | 0.004 | Lower in CKD |
| Chondroitin sulfate proteoglycan 4 | M3WGW7 | 0.004 | Lower in CKD |
| Major prion protein | O18754 | 0.004 | Higher in CKD |
| B-lymphocyte activation antigen B7-1 (CD80) | Q9GMZ8 | 0.004 | Higher in CKD |
| Collagen type XII alpha 1 chain | A0A2I2U432 | 0.004 | Lower in CKD |
| cAMP responsive element binding protein 3 like 3 | M3WK50 | 0.004 | Higher in CKD |
| Cathepsin L | A0A2I2UUI4 | 0.004 | Higher in CKD |
| Copper transport protein ATOX1 | M3W551 | 0.004 | Lower in CKD |
| Polymeric immunoglobulin receptor | M3W5Z6 | 0.004 | Lower in CKD |
| Neural cell adhesion molecule 2 | M3X758 | 0.004 | Lower in CKD |
| Lysyl oxidase homolog | A0A337S0H4 | 0.004 | Lower in CKD |
| Lipocalin/cytosolic fatty-acid binding domain-containing protein | A0A337SF46 | 0.004 | Lower in CKD |
| Ig-like domain-containing protein | A0A5F5XKU3 | 0.004 | Lower in CKD |
| Phosphoinositide-3-kinase-interacting protein 1 | M3VWF4 | 0.004 | Lower in CKD |
| Growth arrest specific 6 | M3W6Q0 | 0.004 | Lower in CKD |
| Beta-hexosaminidase | M3X8Q6 | 0.004 | Higher in CKD |
| Dermokine | A0A5F5XZ81 | 0.004 | Higher in CKD |
| Complement factor B | M3W2B8 | 0.004 | Higher in CKD |
| Galectin-3-binding protein | A0A2I2U679 | 0.004 | Lower in CKD |
| Acid ceramidase | A0A2I2V0N3 | 0.004 | Lower in CKD |
| CD248 molecule | A0A5F5XNG4 | 0.004 | Lower in CKD |
| Immunoglobulin C1-set domain-containing protein | A0A5F5XWQ4 | 0.004 | Higher in CKD |
| Alpha-2-HS-glycoprotein | A0A5F5Y4L1 | 0.004 | Higher in CKD |
| Nidogen 2 | A0A337SMW2 | 0.004 | Higher in CKD |
| Syndecan | M3W3X8 | 0.003 | Lower in CKD |
| Vitronectin | M3W9I5 | 0.003 | Lower in CKD |
| Serpin family F member 1 | M3W5N1 | 0.003 | Higher in CKD |
| Ubiquitin-like domain-containing protein | A0A2I2U2E7 | <0.001 | Lower in CKD |
| Bone marrow stromal cell antigen 2 | A0A2I2U769 | <0.001 | Higher in CKD |
| Albumin | A0A2I2U7Y0 | <0.001 | Higher in CKD |
| Cartilage acidic protein 1 | A0A2I2UDI7 | <0.001 | Lower in CKD |
| T cell immunoglobulin and mucin domain containing 4 | A0A2I2UP29 | <0.001 | Lower in CKD |
| Ig-like domain-containing protein | A0A2I2USM8 | <0.001 | Higher in CKD |
| Angiotensinogen | A0A2I2UUP6 | <0.001 | Higher in CKD |
| ADP-ribosyl cyclase/cyclic ADP-ribose hydrolase | A0A2I2UYP2 | <0.001 | Lower in CKD |
| Endothelial cell adhesion molecule | A0A2I2V4V9 | <0.001 | Lower in CKD |
| Putative protein-lysine deacylase ABHD14B | A0A337RXE3 | <0.001 | Higher in CKD |
| Cell adhesion molecule 1 | A0A337RYW1 | <0.001 | Lower in CKD |
| Leucine rich alpha-2-glycoprotein 1 | A0A337S1P2 | <0.001 | Higher in CKD |
| Ig-like domain-containing protein | A0A337S2C9 | <0.001 | Lower in CKD |
| Cadherin 16 | A0A337S9Q3 | <0.001 | Lower in CKD |
| Ig-like domain-containing protein | A0A337SA19 | <0.001 | Higher in CKD |
| Prolactin-induced protein | A0A337SBR4 | <0.001 | Higher in CKD |
| Limbic system associated membrane protein | A0A337SES8 | <0.001 | Lower in CKD |
| Matrix remodeling-associated protein 8 | A0A337SH59 | <0.001 | Lower in CKD |
| Protein YIPF3 | A0A5F5XCJ6 | <0.001 | Lower in CKD |
| Uncharacterized protein | A0A5F5XFU6 | <0.001 | Higher in CKD |
| Nectin cell adhesion molecule 4 | A0A5F5XLH0 | <0.001 | Lower in CKD |
| MAC-inhibitory protein | A0A5F5XQT8 | <0.001 | Higher in CKD |
| Coagulation factor XII | A0A5F5XVN3 | <0.001 | Higher in CKD |
| Serpin family A member 1 | A0A5F5XW04 | <0.001 | Higher in CKD |
| Serpin family G member 1 | A0A5F5Y090 | <0.001 | Higher in CKD |
| Vitamin K-dependent protein S | A0A5F5Y431 | <0.001 | Lower in CKD |
| Ig-like domain-containing protein | A0A5F5Y438 | <0.001 | Lower in CKD |
| Lipocalin/cytosolic fatty-acid binding domain-containing protein | M3VU75 | <0.001 | Higher in CKD |
| Lymphatic vessel endothelial hyaluronan receptor 1 | M3VUP6 | <0.001 | Lower in CKD |
| EGF containing fibulin extracellular matrix protein 1 | M3VVE6 | <0.001 | Higher in CKD |
| Glutathione hydrolase | M3VWS9 | <0.001 | Lower in CKD |
| Cathepsin S | M3W0Q1 | <0.001 | Higher in CKD |
| Protein FAM151A | M3W1C3 | <0.001 | Lower in CKD |
| Serpin family F member 2 | M3W5N0 | <0.001 | Higher in CKD |
| Dihydrolipoyllysine-residue succinyltransferase component of 2-oxoglutarate dehydrogenase complex, mitochondrial | M3W5T1 | <0.001 | Lower in CKD |
| Serpin family I member 1 | M3W7B6 | <0.001 | Lower in CKD |
| receptor protein-tyrosine kinase | M3W7F9 | <0.001 | Higher in CKD |
| Fibulin 2 | M3W7U0 | <0.001 | Higher in CKD |
| ANTXR cell adhesion molecule 1 | M3W804 | <0.001 | Higher in CKD |
| Fibulin 7 | M3W8Y5 | <0.001 | Lower in CKD |
| Lysosomal alpha-glucosidase | M3W981 | <0.001 | Lower in CKD |
| Dipeptidyl peptidase 1 | M3W9M0 | <0.001 | Lower in CKD |
| Angiopoietin like 2 | M3WA23 | <0.001 | Higher in CKD |
| Macrophage colony-stimulating factor 1 | M3WDW6 | <0.001 | Lower in CKD |

**Supplemental Table S6.** Receiver operating characteristic curve for CKD diagnosis in test cohort.

| **Urinary protein** | **SingleID** | **Area under curve [95% CI]** |
| --- | --- | --- |
| Retinol binding protein 4 | M5AXY1 | 0.957 [0.885, 1] |
| Gelsolin | A0A5F5XIG6 | 0.91 [0.825, 0.996] |
| ADF-H domain-containing protein | A0A5F5XRM0 | 0.895 [0.794, 0.996] |
| IaI heavy chain 4 | A0A5F5Y7V2 | 0.895 [0.796, 0.994] |
| Secreted phosphoprotein 1 | A0A2I2UVQ2 | 0.877 [0.772, 0.983] |
| Ig-like domain-containing protein | M3X4T8 | 0.875 [0.771, 0.979] |
| Fel d 7 allergen | E5D2Z5 | 0.849 [0.721, 0.977] |
| Antithrombin-III | M3WLL8 | 0.847 [0.715, 0.978] |
| Transferrin | M3X260 | 0.836 [0.703, 0.97] |
| Kinesin-like protein | A0A5F5XE91 | 0.834 [0.692, 0.976] |
| Clusterin | M3WKP2 | 0.831 [0.7, 0.963] |
| Superoxide dismutase [Cu-Zn] | A0A2I2UPH5 | 0.829 [0.695, 0.962] |
| Carboxylic ester hydrolase | M3W105 | 0.826 [0.685, 0.967] |
| Fatty acid-binding protein, intestinal | M3WQY4 | 0.816 [0.68, 0.952] |
| Alpha-amylase | M3X5H0 | 0.816 [0.676, 0.956] |
| Prostaglandin-H2 D-isomerase | Q29487 | 0.813 [0.668, 0.958] |
| Ig-like domain-containing protein | A0A337S7W6 | 0.808 [0.66, 0.956] |
| Transthyretin | M3WEV9 | 0.788 [0.64, 0.936] |
| Serpin family F member 1 | M3W5N1 | 0.785 [0.641, 0.93] |
| Ig-like domain-containing protein | A0A337SA19 | 0.777 [0.625, 0.93] |
| Syndecan | M3W3X8 | 0.772 [0.626, 0.919] |
| Vascular cell adhesion molecule 1 | M3WED4 | 0.772 [0.622, 0.923] |
| Anaphylatoxin-like domain-containing protein | A0A5F5XM97 | 0.77 [0.615, 0.925] |
| Nidogen 1 | M3WED3 | 0.77 [0.62, 0.92] |
| Thy-1 membrane glycoprotein | A0A2I2UGV7 | 0.767 [0.613, 0.922] |
| Cystatin E/M | M3WIK5 | 0.762 [0.61, 0.915] |
| Albumin | A0A2I2U7Y0 | 0.757 [0.597, 0.918] |
| Albumin | P49064 | 0.757 [0.607, 0.907] |
| Desmocollin 1 | A0A2I2UZT4 | 0.749 [0.597, 0.902] |
| Plasminogen | M3X3T9 | 0.747 [0.582, 0.911] |
| CXADR Ig-like cell adhesion molecule | A0A337S2K2 | 0.744 [0.579, 0.909] |
| Cadherin 13 | M3W235 | 0.744 [0.584, 0.904] |
| Myelin protein zero like 2 | M3WFQ7 | 0.739 [0.579, 0.9] |
| Ganglioside GM2 activator | M3WHT1 | 0.739 [0.581, 0.897] |
| Metalloendopeptidase | M3WB64 | 0.734 [0.579, 0.889] |
| Ig-like domain-containing protein | A0A2I2USM8 | 0.721 [0.558, 0.884] |
| Collagen type XVIII alpha 1 chain | A0A5F5XC75 | 0.716 [0.549, 0.884] |
| Ig-like domain-containing protein | A0A5F5XZ27 | 0.714 [0.542, 0.885] |
| N-acetylglucosamine-6-sulfatase | M3VUR6 | 0.714 [0.545, 0.883] |
| Cystatin C | D5MTH1 | 0.711 [0.547, 0.875] |
| Ig-like domain-containing protein | A0A337SA21 | 0.708 [0.544, 0.873] |
| MANSC domain containing 1 | A0A337SHI7 | 0.703 [0.539, 0.868] |
| Putative protein-lysine deacylase ABHD14B | A0A337RXE3 | 0.701 [0.527, 0.875] |
| Nidogen 2 | A0A337SMW2 | 0.698 [0.532, 0.864] |
| Aminopeptidase | M3VU18 | 0.698 [0.532, 0.865] |
| Serpin family A member 1 | A0A5F5XW04 | 0.696 [0.525, 0.866] |
| Hemicentin 2 | M3X7U8 | 0.691 [0.523, 0.858] |
| Coagulation factor XII | A0A5F5XVN3 | 0.683 [0.513, 0.853] |
| Ig-like domain-containing protein | A0A337S2C9 | 0.68 [0.503, 0.857] |
| Cystatin domain-containing protein | A0A337SE08 | 0.68 [0.512, 0.849] |
| ADP-ribosyl cyclase/cyclic ADP-ribose hydrolase | A0A2I2UYP2 | 0.678 [0.507, 0.848] |
| Afamin | A0A2I2U1Z3 | 0.673 [0.498, 0.847] |
| Fibulin 2 | M3W7U0 | 0.662 [0.487, 0.838] |
| Ig-like domain-containing protein | A0A5F5XP19 | 0.657 [0.487, 0.827] |
| Adiposis regulatory factor | M3W8Q3 | 0.657 [0.482, 0.833] |
| Immunoglobulin superfamily member 8 | A0A2I2U7U8 | 0.652 [0.46, 0.844] |
| Angiopoietin like 3 | M3WET8 | 0.652 [0.479, 0.825] |
| Collectin-12 | M3WN82 | 0.652 [0.474, 0.83] |
| Fibrillin 1 | M3WMG2 | 0.645 [0.469, 0.82] |
| Heparan sulfate proteoglycan 2 | A0A5F5Y6R8 | 0.639 [0.461, 0.818] |
| ANTXR cell adhesion molecule 1 | M3W804 | 0.639 [0.461, 0.818] |
| Dermatopontin | M3XE34 | 0.637 [0.457, 0.817] |
| Fibrinogen alpha chain | M3W022 | 0.634 [0.46, 0.809] |
| Heteroous nuclear ribonucleoprotein M | A0A5F5XQH4 | 0.629 [0.45, 0.808] |
| Complement factor B | M3W2B8 | 0.629 [0.446, 0.812] |
| Vitamin K-dependent protein S | A0A5F5Y431 | 0.627 [0.446, 0.807] |
| Dipeptidyl peptidase 1 | M3W9M0 | 0.627 [0.449, 0.804] |
| Junctional adhesion molecule A | Q2WGK2 | 0.619 [0.442, 0.796] |
| Dystroglycan 1 | A0A337SFQ4 | 0.616 [0.439, 0.794] |
| Desmocollin 2 | A0A5F5XHZ3 | 0.616 [0.433, 0.8] |
| Lectin, mannose binding 2 | M3XF94 | 0.616 [0.436, 0.797] |
| Ig-like domain-containing protein | A0A2I2U3F6 | 0.611 [0.434, 0.788] |
| Lymphocyte antigen 6 family member D | A0A337SJ42 | 0.609 [0.421, 0.796] |
| cAMP responsive element binding protein 3 like 3 | M3WK50 | 0.609 [0.416, 0.802] |
| Peptidase S1 domain-containing protein | M3X005 | 0.609 [0.429, 0.788] |
| Lipocalin/cytosolic fatty-acid binding domain-containing protein | M3WIS7 | 0.606 [0.428, 0.785] |
| Lipocalin/cytosolic fatty-acid binding domain-containing protein | M3VU75 | 0.604 [0.418, 0.79] |
| EGF containing fibulin extracellular matrix protein 1 | M3VVE6 | 0.598 [0.418, 0.779] |
| Chromogranin-A | A0A5F5XNS6 | 0.591 [0.407, 0.775] |
| alpha-1,2-Mannosidase | A0A5F5Y426 | 0.591 [0.398, 0.784] |
| receptor protein-tyrosine kinase | M3W7F9 | 0.591 [0.404, 0.777] |
| SH3 domain-binding glutamic acid-rich-like protein 3 | M3WHM8 | 0.591 [0.408, 0.773] |
| Cathepsin S | M3W0Q1 | 0.588 [0.405, 0.771] |
| Prolactin-induced protein | A0A337SBR4 | 0.583 [0.393, 0.773] |
| Inter-alpha-trypsin inhibitor heavy chain 5 | M3WEM7 | 0.581 [0.397, 0.764] |
| Cadherin 11 | M3W046 | 0.578 [0.392, 0.764] |
| Fibulin-5 | M3WFS9 | 0.578 [0.395, 0.761] |
| Fibulin 7 | M3W8Y5 | 0.573 [0.387, 0.759] |
| Carboxylesterase 5A | Q8I034 | 0.57 [0.38, 0.761] |
| Angiotensinogen | A0A2I2UUP6 | 0.568 [0.383, 0.753] |
| Basigin | A0A5F5Y008 | 0.565 [0.377, 0.753] |
| Cadherin-1 | A0A337SG59 | 0.563 [0.373, 0.752] |
| Chymotrypsin C | M3W019 | 0.563 [0.38, 0.746] |
| Insulin-like growth factor-binding protein 3 | A0A337S9R3 | 0.555 [0.373, 0.737] |
| Filamin A | A0A2I2V3V8 | 0.552 [0.366, 0.738] |
| LY6/PLAUR domain containing 2 | M3VU52 | 0.552 [0.363, 0.742] |
| Restriction factor for feline retrovirus X | T2HUV9 | 0.552 [0.371, 0.734] |
| Dermokine | A0A5F5XZ81 | 0.547 [0.353, 0.741] |
| Proheparin-binding EGF-like growth factor | M3VWC1 | 0.547 [0.351, 0.744] |
| Angiopoietin like 2 | M3WA23 | 0.547 [0.358, 0.737] |
| Corneodesmosin | M3X143 | 0.537 [0.354, 0.721] |
| Major allergen I polypeptide chain 1 | P30438 | 0.527 [0.338, 0.716] |
| Hemicentin 1 | M3X1M2 | 0.522 [0.335, 0.709] |
| Leucine rich alpha-2-glycoprotein 1 | A0A337S1P2 | 0.519 [0.336, 0.703] |
| B-lymphocyte activation antigen B7-1 (CD80) | Q9GMZ8 | 0.512 [0.329, 0.694] |
| LIM and SH3 domain protein 1 | M3X768 | 0.496 [0.31, 0.682] |
| Cathelicidin | F1C962 | 0.491 [0.299, 0.683] |
| Cathepsin L | A0A2I2UUI4 | 0.488 [0.3, 0.677] |
| Serpin family G member 1 | A0A5F5Y090 | 0.488 [0.304, 0.673] |
| Lipocalin like 1 | M3XCK7 | 0.488 [0.305, 0.672] |
| Uncharacterized protein | A0A5F5XFU6 | 0.483 [0.298, 0.669] |
| Proenkephalin-A | A0A5F5XWQ5 | 0.483 [0.299, 0.668] |
| Elastase, neutrophil expressed | M3XDQ5 | 0.445 [0.263, 0.627] |
| Bone marrow stromal cell antigen 2 | A0A2I2U769 | 0.44 [0.257, 0.622] |
| Major prion protein | O18754 | 0.435 [0.247, 0.622] |

**Supplemental Table S7.** The subset of proteins that were selected by elastic net regression and included in the additional analysis.

| **UniProt ID** | **Weight** | **Protein** |
| --- | --- | --- |
| Q8I034 | 0.1550 | Carboxylesterase 5A |
| A0A2I2UUI4 | 0.1501 | Cathepsin L |
| M3WIK5 | 0.1468 | Cystatin E/M |
| M3VU52 | 0.1453 | LY6/PLAUR domain containing 2 |
| M3W235 | 0.1444 | Cadherin 13 |
| A0A2I2UZT4 | 0.1399 | Desmocollin 1 |
| A0A2I2V3V8 | 0.1347 | Filamin A |
| A0A5F5XE91 | 0.1336 | Kinesin-like protein |
| A0A2I2UVQ2 | 0.1237 | Secreted phosphoprotein 1 |
| M3X768 | 0.1230 | LIM and SH3 domain protein 1 |
| A0A2I2U7U8 | 0.1209 | Immunoglobulin superfamily member 8 |
| P30438 | 0.1072 | Major allergen I polypeptide chain 1 |
| A0A2I2UGV7 | 0.1048 | Thy-1 membrane glycoprotein |
| A0A337SG59 | 0.1001 | Cadherin-1 |
| M3WMG2 | 0.0994 | Fibrillin 1 |
| A0A5F5XNS6 | 0.0990 | Chromogranin-A |
| A0A5F5Y6R8 | 0.0920 | Heparan sulfate proteoglycan 2 |
| M3W3X8 | 0.0885 | Syndecan |
| E5D2Z5 | 0.0853 | Fel d 7 allergen |
| M3X4T8 | 0.0775 | Ig-like domain-containing protein |
| M3X1M2 | 0.0768 | Hemicentin 1 |
| A0A5F5XRM0 | 0.0756 | ADF-H domain-containing protein |
| M3X3T9 | 0.0721 | Plasminogen |
| M3XF94 | 0.0710 | Lectin, mannose binding 2 |
| M3WIS7 | 0.0709 | Lipocalin/cytosolic fatty-acid binding domain-containing protein |
| M3X143 | 0.0698 | Corneodesmosin |
| A0A337S2K2 | 0.0679 | CXADR Ig-like cell adhesion molecule |
| M3VWC1 | 0.0671 | Proheparin-binding EGF-like growth factor |
| A0A2I2U1Z3 | 0.0603 | Afamin |
| A0A5F5XIG6 | 0.0566 | Gelsolin |
| M3WED3 | 0.0557 | Nidogen 1 |
| M3WB64 | 0.0543 | Metalloendopeptidase |
| A0A5F5XW04 | 0.0539 | Serpin family A member 1 |
| M3W105 | 0.0470 | Carboxylic ester hydrolase |
| M3WQY4 | 0.0433 | Fatty acid-binding protein, intestinal |
| M3VU18 | 0.0409 | Aminopeptidase |
| A0A337SMW2 | 0.0403 | Nidogen 2 |
| M3W8Y5 | 0.0379 | Fibulin 7 |
| A0A5F5XZ27 | 0.0348 | Ig-like domain-containing protein |
| M3WKP2 | 0.0336 | Clusterin |
| D5MTH1 | 0.0315 | Cystatin C |
| M3W046 | 0.0305 | Cadherin 11 |
| M3W1C3 | 0.0291 | Protein FAM151A |
| M3X5H0 | 0.0283 | Alpha-amylase |
| M3WFS9 | 0.0268 | Fibulin-5 |
| A0A337SA19 | 0.0253 | Ig-like domain-containing protein |
| M3WHT1 | 0.0235 | Ganglioside GM2 activator |
| A0A5F5Y008 | 0.0231 | Basigin |
| A0A5F5Y7V2 | 0.0218 | IaI heavy chain 4 |
| M3WEM7 | 0.0216 | Inter-alpha-trypsin inhibitor heavy chain 5 |
| M3X7U8 | 0.0207 | Hemicentin 2 |
| M3WED4 | 0.0178 | Vascular cell adhesion molecule 1 |
| M3WHM8 | 0.0171 | SH3 domain-binding glutamic acid-rich-like protein 3 |
| M3WFQ7 | 0.0155 | Myelin protein zero like 2 |
| A0A2I2UUP6 | 0.0139 | Angiotensinogen |
| A0A5F5XC75 | 0.0134 | Collagen type XVIII alpha 1 chain |
| Q29487 | 0.0108 | Prostaglandin-H2 D-isomerase |
| A0A337S9R3 | 0.0088 | Insulin-like growth factor-binding protein 3 |
| M3W8Q3 | 0.0076 | Adiposis regulatory factor |
| A0A2I2USM8 | 0.0029 | Ig-like domain-containing protein |
| M3X260 | 0.0029 | Transferrin |
| M3WN82 | 0.0006 | Collectin-12 |
| A0A5F5XP19 | -0.0012 | Ig-like domain-containing protein |
| M3XBL5 | -0.0017 | Joining chain of multimeric IgA and IgM |
| A0A2I2UAM6 | -0.0049 | Aquaporin-1 |
| M3WJA1 | -0.0061 | Pepsin A |
| A0A5F5XWQ4 | -0.0078 | Immunoglobulin C1-set domain-containing protein |
| M3WKI3 | -0.0082 | Cartilage intermediate layer protein 2 |
| A0A5F5XI37 | -0.0100 | Lipocalin/cytosolic fatty-acid binding domain-containing protein |
| M3W2Z1 | -0.0107 | Vitamin D-binding protein |
| A0A5F5Y466 | -0.0153 | Cadherin related family member 5 |
| A0A5F5XVF5 | -0.0183 | Kininogen 1 |
| M3XC02 | -0.0189 | Dimethylarginine dimethylaminohydrolase 2 |
| M3W064 | -0.0196 | Nucleobindin-1 |
| A0A337SH59 | -0.0221 | Matrix remodeling-associated protein 8 |
| Q5MGS7 | -0.0231 | Beta-2-microglobulin |
| M3WX61 | -0.0237 | Ribonuclease A family member k6 |
| A0A337S9Q3 | -0.0245 | Cadherin 16 |
| M3W5B5 | -0.0250 | LDL receptor related protein 2 |
| A0A5F5Y4L1 | -0.0252 | Alpha-2-HS-glycoprotein |
| Q9GMZ8 | -0.0284 | B-lymphocyte activation antigen B7-1 (CD80) |
| A0A5F5Y3H5 | -0.0344 | Transmembrane protein 277 |
| M3WSA1 | -0.0347 | Gamma-interferon-inducible lysosomal thiol reductase |
| M3W2U8 | -0.0396 | Basal cell adhesion molecule (Lutheran blood group) |
| M3W4L2 | -0.0401 | Insulin-like growth factor-binding protein 1 |
| M3WSF5 | -0.0423 | Solute carrier family 51 subunit beta |
| A0A337SS27 | -0.0456 | Aminopeptidase N |
| A0A2I2U5M6 | -0.0459 | Ig-like domain-containing protein |
| M3WNU0 | -0.0494 | Interleukin 1 receptor accessory protein |
| A0A5F5Y312 | -0.0497 | Stratifin |
| M3X7A8 | -0.0512 | Ubiquitin like 7 |
| M3VU75 | -0.0513 | Lipocalin/cytosolic fatty-acid binding domain-containing protein |
| A0A0A0MPZ5 | -0.0522 | Glyceraldehyde-3-phosphate dehydrogenase |
| A0A2I2UF36 | -0.0525 | Interleukin 18 binding protein |
| M3WQA9 | -0.0533 | Sulfotransferase |
| M3W0W4 | -0.0596 | Alpha-1-B glycoprotein |
| A0A5F5XVS8 | -0.0604 | Dipeptidyl peptidase 7 |
| A0A2I2UPA5 | -0.0616 | NHL repeat containing 3 |
| P07412 | -0.0623 | Hemoglobin subunit beta-A/B |
| A0A2I2U1V5 | -0.0646 | Alpha-galactosidase |
| M3WCX0 | -0.0648 | Corticosteroid-binding globulin |
| M3X0F5 | -0.0659 | Nuclear transport factor 2 |
| M3WAQ7 | -0.0664 | Hexosyltransferase |
| A0A337S474 | -0.0704 | Urokinase-type plasminogen activator |
| A0A2I2UVC1 | -0.0734 | Integral membrane protein 2 |
| M3WPP7 | -0.0740 | Maltase-glucoamylase |
| M3W5N0 | -0.0759 | Serpin family F member 2 |
| M3VVI7 | -0.0760 | cathepsin X |
| A0A337SMK2 | -0.0795 | CD44 antigen |
| A0A337S0Y0 | -0.0809 | Sulfurtransferase |
| M3XEW0 | -0.0891 | Renin receptor |
| M3WSI8 | -0.0918 | Prothrombin |
| M3XC93 | -0.0952 | Actin, alpha skeletal muscle |
| M3WXF2 | -0.0953 | Peptidase inhibitor 16 |
| M3W8S0 | -0.0960 | Ezrin |
| M3WEQ2 | -0.0972 | Vasorin |
| M3WPG6 | -0.0988 | Apolipoprotein A-I |
| A0A2I2U3H1 | -0.0995 | Insulin like growth factor binding protein 7 |
| A0A337S785 | -0.1008 | Trans-1,2-dihydrobenzene-1,2-diol dehydrogenase |
| M3VYA2 | -0.1027 | Protein C receptor |
| A0A337S3R0 | -0.1171 | Sushi domain-containing protein |
| E3UTY9 | -0.1175 | Haptoglobin |
| A0A2I2USC5 | -0.1224 | EMI domain containing 1 |
| M3WI47 | -0.1254 | Prosaposin |
| A0A2I2UBZ5 | -0.1275 | PDZ domain containing 1 |
| A0A5F5XWB9 | -0.1403 | Pentraxin family member |
| A0A2I2UP29 | -0.1473 | T cell immunoglobulin and mucin domain containing 4 |
| A0A2I2UL25 | -0.1479 | Peroxiredoxin-6 |
| M3W6I2 | -0.1538 | Actin, cytoplasmic 2 |
| A0A2I2V455 | -0.1568 | N-acylethanolamine-hydrolyzing acid amidase |
| M3X315 | -0.1573 | Immunoglobulin superfamily containing leucine rich repeat |
| M3WJ25 | -0.1614 | Cubilin |
| A0A5F5XPV2 | -0.1625 | Lipase |
| A0A5F5Y096 | -0.1642 | MBL associated serine protease 2 |
| A0A2I2UTQ4 | -0.1724 | receptor protein-tyrosine kinase |
